# Microglial phagocytosis of living photoreceptors contributes to inherited retinal degeneration

**DOI:** 10.15252/emmm.201505298

**Published:** 2015-07-02

**Authors:** Lian Zhao, Matthew K Zabel, Xu Wang, Wenxin Ma, Parth Shah, Robert N Fariss, Haohua Qian, Christopher N Parkhurst, Wen-Biao Gan, Wai T Wong

**Affiliations:** 1 Unit on Neuron-Glia Interactions in Retinal Disease, National Eye institute, National Institutes of HealthBethesda, MD, USA; 2 Biological Imaging Core, National Eye institute, National Institutes of HealthBethesda, MD, USA; 3 Visual Function Core, National Eye institute, National Institutes of HealthBethesda, MD, USA; 4 Department of Neuroscience and Physiology, Skirball Institute, New York University School of MedicineNew York, NY, USA

**Keywords:** apoptosis, microglia, phagocytosis, retinal degeneration, retinitis pigmentosa

## Abstract

Retinitis pigmentosa, caused predominantly by mutations in photoreceptor genes, currently lacks comprehensive treatment. We discover that retinal microglia contribute non-cell autonomously to rod photoreceptor degeneration by primary phagocytosis of living rods. Using rd10 mice, we found that the initiation of rod degeneration is accompanied by early infiltration of microglia, upregulation of phagocytic molecules in microglia, and presentation of “eat-me” signals on mutated rods. On live-cell imaging, infiltrating microglia interact dynamically with photoreceptors via motile processes and engage in rapid phagocytic engulfment of non-apoptotic rods. Microglial contribution to rod demise is evidenced by morphological and functional amelioration of photoreceptor degeneration following genetic ablation of retinal microglia. Molecular inhibition of microglial phagocytosis using the vitronectin receptor antagonist cRGD also improved morphological and functional parameters of degeneration. Our findings highlight primary microglial phagocytosis as a contributing mechanism underlying cell death in retinitis pigmentosa and implicate microglia as a potential cellular target for therapy.

## Introduction

Retinitis pigmentosa (RP) is a set of blinding hereditary retinal diseases affecting an estimated one million patients worldwide in which, typically rod, then cone, photoreceptors, degenerate as a result of mutations predominantly in genes expressed in photoreceptors or retinal pigment epithelial (RPE) cells (Hartong *et al*, 2006). Although gene therapy approaches to correct individual mutations hold promise (Maguire *et al*, 2008), the significant number and diversity of causative genes (> 100) (Daiger *et al*, 2013) underscores the need to identify and target broadly shared mechanisms. Clinical and laboratory findings indicate that in addition to cell-autonomous changes arising directly from mutations in photoreceptor-expressed genes (Sancho-Pelluz *et al*, 2008), non-cell-autonomous mechanisms involving inflammation (Mustafi *et al*, 2012; Yoshida *et al*, 2013a) may contribute to photoreceptor degeneration. Microglia, the resident inflammatory cells in the retina, normally excluded from the outer nuclear layer (ONL), but which migrate into close proximity to photoreceptors in animal models (Roque *et al*, 1996) and human specimens (Gupta *et al*, 2003) of RP, are thought to induce degenerative changes. Suppression of the activation (Peng *et al*, 2014) and pro-oxidative properties (Yoshida *et al*, 2013b; Zeng *et al*, 2014) of microglia appear to alleviate retinal degeneration in rodent models; however, the cellular mechanisms whereby retinal microglia interact with stressed photoreceptors to induce their demise remain undefined.

Recent evidence has highlighted the role of CNS microglia not only in removing debris and apoptotic neurons, but also in executing neuronal death via the phagocytosis of stressed but living neurons (Brown & Neher, 2014). This process of primary phagocytosis, also termed “phagoptosis” (Brown & Neher, 2012), has been found to be a primary cause of neuronal cell death in the developing CNS (Cunningham *et al*, 2013) and in animal models of stroke (Neher *et al*, 2013) and neuroinflammation (Fricker *et al*, 2012). However, its presence and participation in retinal disease is unknown. We examined interactions between retinal microglia and photoreceptors at each stage of degeneration to discover cellular mechanisms underlying microglia-mediated photoreceptor death. Using the rd10 mouse model of RP induced by a mutation in the rod photoreceptor-specific Pde6b gene (Chang *et al*, 2007), a causative gene in human RP (McLaughlin *et al*, 1993), we demonstrate that microglia infiltrate the ONL early during degeneration and phagocytose non-apoptotic, living rod photoreceptors via a process of dynamic physical contact followed by rapid engulfment and internalization. We found that the primary phagocytosis in the ONL corresponded to an upregulation of phagocytic molecules in microglia, and a concurrent exposure of the “eat-me” signal, phosphatidylserine, in stressed rods. The non-cell-autonomous contribution of microglia is evidenced by the morphological and functional amelioration of photoreceptor degeneration upon the genetic ablation of retinal microglia and the molecular inhibition of microglial phagocytosis, indicating that microglial phagocytosis of living rods contributes to inherited photoreceptor degeneration. Additionally, activated infiltrating microglia can also potentiate rod apoptosis via the secretion of IL-1β, which further accelerates degeneration via pro-inflammatory mechanisms. These non-cell-autonomous microglia-associated factors that exacerbate rod degeneration highlight the opportunities to ameliorate photoreceptor loss and vision loss in RP via microglia-directed therapies.

## Results

### Microglial infiltration and morphological change during rod degeneration

**Figure 1:**
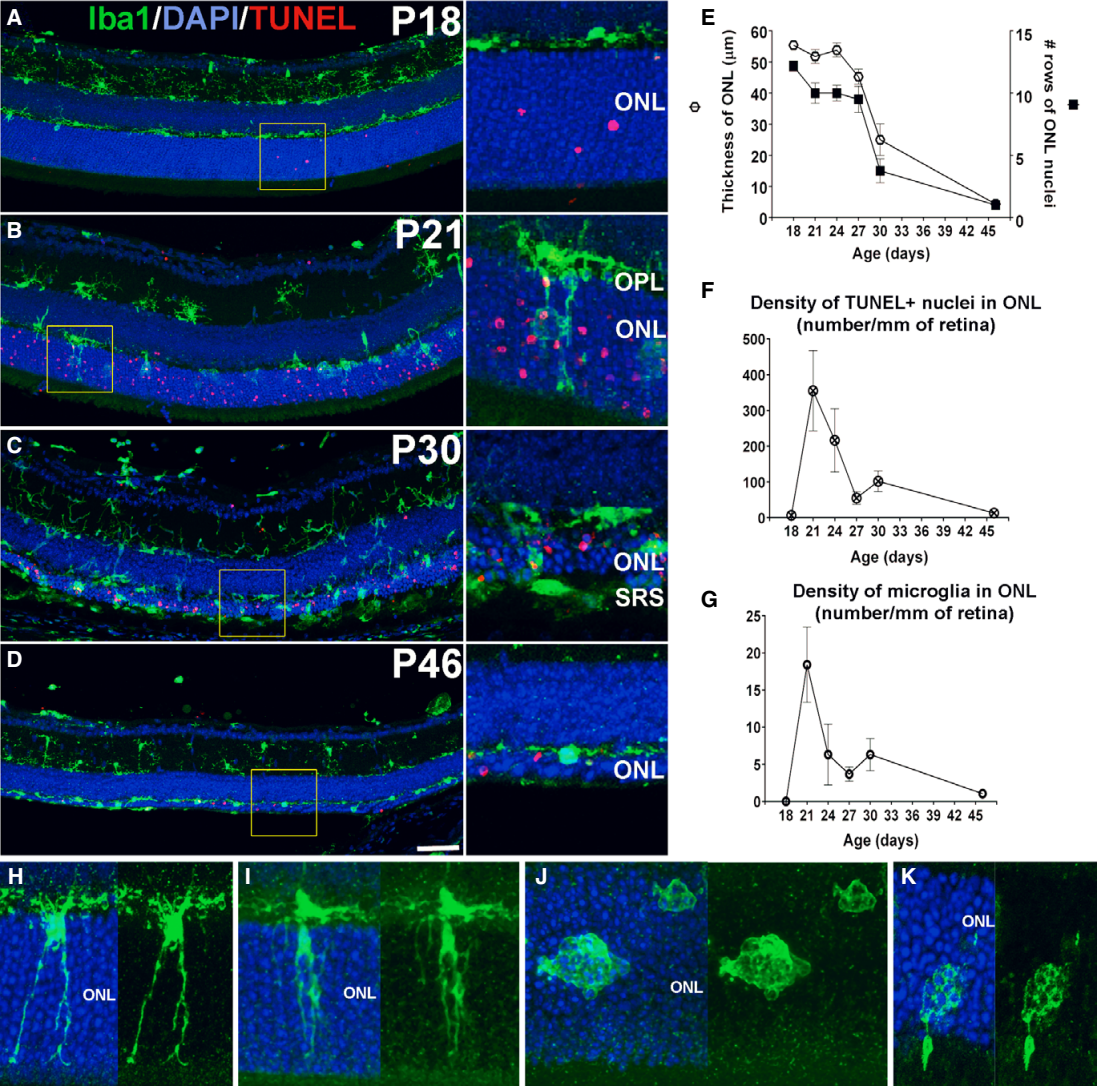
Early translocation of retinal microglia from the inner to the outer nuclear layer (ONL) during rod degeneration in rd10 mice A–D Progression of microglial infiltration in the ONL. Early in the course of rod degeneration at P18, few isolated apoptotic TUNEL-positive nuclei (red, inset) were present in the ONL, with Iba1^+^ microglial somata and processes (green) confined to the inner retina (A). At P21, microglia in the outer plexiform layer (OPL) extended radial processes into the ONL (inset), with a concurrent increase in the number of TUNEL^+^ nuclei (B). At P30, retinal microglia infiltrated the entire depth of the markedly thinned ONL and the subretinal space (SRS) (inset) (C). At P46, microglia became less dense in the ONL and reverted to a more ramified morphology (D). Scale bar, 50 μm.

We monitored the distribution and morphology of retinal microglia across rod degeneration in the rd10 mouse (Chang *et al*, 2007). At P18, prior to the onset of atrophy, horizontally ramified microglia were limited to the inner retina in layers vitreal to the outer nuclear layer (ONL); only a few isolated apoptotic (TUNEL^+^) nuclei were present in the ONL (Fig1A). At P21, as the number of TUNEL^+^ nuclei increased in the ONL, microglia infiltrated the ONL via radially oriented cellular processes and by cellular migration (Fig1B). At P30, as the ONL progressively thinned, the densities of infiltrating microglia and TUNEL^+^ nuclei in the ONL declined (Fig1C), falling to low levels by P46 (Fig1D and E). Quantitative analysis demonstrated that during this time, the processes of rod apoptosis (quantitated as the density of TUNEL^+^ nuclei) and microglial infiltration displayed similar temporal patterns, peaking at P21 and diminishing thereafter (Fig1F and G). Microglia infiltrating the ONL acquired morphological features absent in inner retinal microglia, including: a redirection of processes to a predominantly radial orientation, extension of processes across the ONL to intercalate closely with photoreceptor somata (Fig1H), development of intracellular phagosomes in cellular processes (Fig1I), and acquisition of a rounded, amoeboid morphology containing multiple phagosomes (Fig1J and K).

E–G Quantitative analyses demonstrate that ONL atrophy progression, characterized by ONL thickness (empty symbols*)* and number of rows of ONL nuclei (filled symbols*)* (E), occurred concurrently with the progression of ONL apoptosis, followed as the density of TUNEL^+^ nuclei in the ONL (F), and microglia infiltration, followed as microglial density in the ONL (G) (*n* = 3 animals at each time point). Data points and error bars indicate mean ± SEM.

H–K Higher magnification images showing microglial process infiltration (H), phagosome formation in infiltrating processes (I), and microglial transformation into amoeboid morphologies containing multiple phagosomes (J,K).

### Transient expression of phagocytic molecules in the outer nuclear layer during rod degeneration

**Figure EV1:**
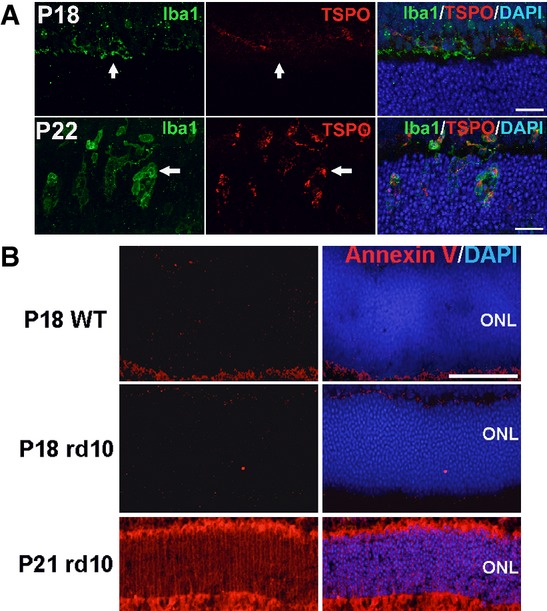
Activation status of microglia infiltrating the outer nuclear layer (ONL) and the exposure of phosphatidylserine (PS) on ONL photoreceptors Microglia infiltrating the outer nuclear layer (ONL) of the rd10 retina during rod photoreceptor degeneration demonstrate markers of activation. At P18, Iba1^+^ microglia (green, arrow) in the outer plexiform layer were negative for TSPO (red), an activation marker. At P22, Iba1^+^ microglia (arrow) infiltrated the ONL and acquired TSPO immunopositivity, indicating their activated status. Scale bars, 25 μm.

**Figure 2:**
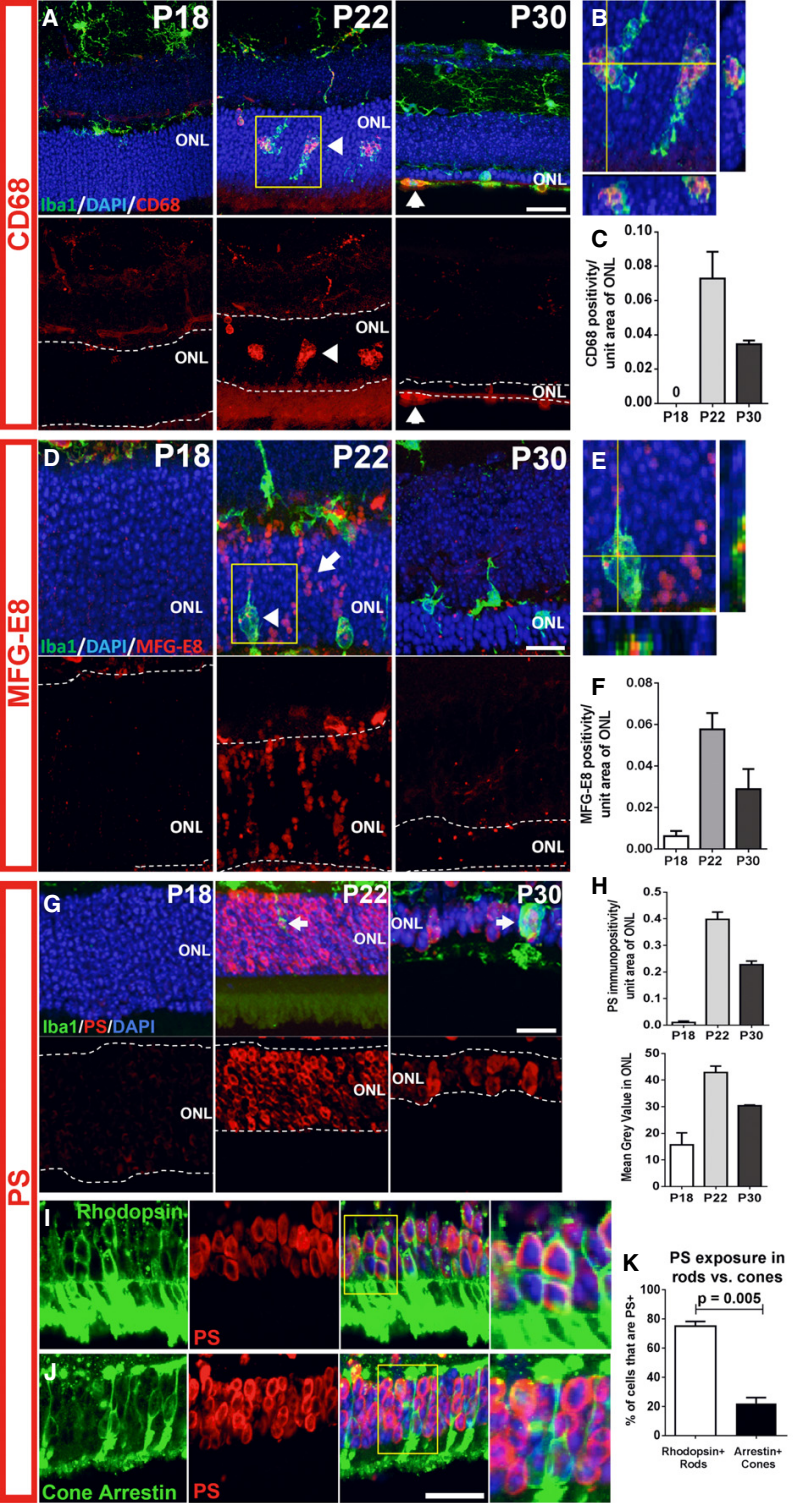
Transient expression of phagocytic molecules and exposure of the “eat-me” signal phosphatidylserine (PS) in the outer nuclear layer during rod degeneration A–C Microglia infiltrating the ONL demonstrate upregulation of the phagocytic molecule, CD68. Top images in (A) show composites of CD68 (red), Iba1 (green), and DAPI (blue) staining; bottom images show the same field with CD68 staining only. At P18, non-infiltrating microglia expressed low or undetectable levels of CD68, a lysosome-associated membrane protein. At P21–23, microglia infiltrating the ONL strongly upregulated CD68 (arrowheads). At P30, CD68 immunopositivity in ONL microglia decreased and was mostly confined to amoeboid cells in the subretinal space. (B) Magnified view of inset in (A) demonstrates localization of CD68 to microglia phagosomes. (C) CD68 expression (area of immunopositivity as a fraction of the ONL) demonstrated a peaked at P22 and decreased by P30. Scale bar, 20 μm.

As the morphological changes in infiltrating microglia suggested phagocytic function, we examined microglia for the expression of molecular markers of phagocytosis by immunohistochemistry. CD68, a lysosome-associated membrane protein (LAMP) and scavenger receptor (Holness *et al*, 1993), and a marker for activated phagocytic monocytic cells (Graeber *et al*, 1990), was expressed at very low levels prior to microglial infiltration at P18 (Fig2A), but increased prominently in the ONL at P21–22, co-localizing to Iba1^+^ infiltrating microglia, specifically to microglial phagosomes (Fig2B), before diminishing at P30 (Fig2C). This indicated that the vesicular structures located in ONL microglia were functional phagosomes. Immunopositivity for lactadherin/milk fat globule-EGF factor 8 protein (MFG-E8), a microglial-secreted adaptor protein that acts as a “bridging” molecule between the vitronectin receptor on phagocytes and phosphatidylserine (PS) on phagocytosed neurons (Fuller & Van Eldik, 2008; Neniskyte & Brown, 2013), was low or absent in the ONL at P18 (Fig2D), but emerged prominently at P21–22, localizing to infiltrating microglia (Fig2E), and to the surfaces of ONL photoreceptors, before decreasing at P30 (Fig2F). These observations indicate that microglia transiently upregulate phagocytic and lysosomal function upon ONL infiltration in a temporal pattern parallel to rod degeneration. The immunopositivity of CD68 in infiltrating microglia was correlated with that of translocator protein (TSPO), a biomarker for microglial activation (Wang *et al*, 2014), indicating increased activation of infiltrating microglia (Fig EV1A).

Phosphatidylserine (PS) exposure in ONL nuclei of unfixed cryosections of rd10 retina during rod degeneration. PS exposure in the ONL was monitored in unfixed frozen sections using fluorescently conjugated annexin V which binds cell-surface PS. While minimal annexin V staining was evident in the ONL in P18 wild-type (top row) and P18 rd10 (middle row) retinas in which rod degeneration is absent, staining was prominent in P21 rd10 retina during rod degeneration (bottom row). Scale bar, 40 μm.

D–F Upregulation of MFG-E8, a secreted glycoprotein that serves as a bridging molecule for phagocytosis receptors, in the ONL. (D) MFG-E8 was low or absent in the ONL at P18, but emerged at P21–23, localizing to photoreceptor cell bodies in a column-like pattern (arrow) and as a punctate signal within infiltrating microglia (arrowhead), before decreasing throughout the ONL at P30. (E) A magnified orthogonal view of the inset from (D) demonstrating punctate MFG-E8 immunopositivity within ONL microglia. (F) Quantification of MFG-E8 expression demonstrated a prominent emergence at P22 and a subsequent decrement by P30. Scale bar, 20 μm.

G–K Increase in phosphatidylserine (PS) exposure in the ONL during photoreceptor degeneration. (G) At P18, PS immunopositivity is near absent in the ONL, but increased significantly in ONL somata at P21–23, before decreasing at P30. (H) Quantitation of PS exposure by image analysis (by fractional area of PS immunopositivity within the ONL (top), and the mean intensity of PS staining in the ONL (bottom)) demonstrated a transient increase at P22. (I) Co-immunolabeling of rods with rhodopsin at P22 demonstrates that PS exposure was present in a majority of rods (inset shows at high magnification the co-labeling of PS and rhodopsin in multiple rod somata). (J) Conversely, immunolabeling of cones with cone arrestin demonstrates the sparse co-localization of PS in cones (inset shows close juxtaposition but no colocalization of PS and arrestin labeling). (K) Scoring of rhodopsin^+^ rods and arrestin^+^ cones for PS co-labeling demonstrates that a large majority of rods, but only a small minority of cones, showed PS exposure (two-sided unpaired *t*-test, *n* = 3 animals at P22). Scale bars = 20 μm. Data information: Quantitative analyses in (C, F, H, and K) involved three animals at each time point. Column heights and error bars indicate mean ± SEM.

To explore the emergence of cellular targets for infiltrating phagocytic microglia, we examined the ONL for the exposure of phosphatidylserine (PS), an “eat-me” signal recognized by engulfment receptors on phagocytes during phagocytosis initiation (Ravichandran, 2011). Exposure of PS, which was low or absent in the ONL at P18, developed in multiple ONL nuclei at P22, and declined at P30 (Fig2G), as demonstrated by quantifying the fractional occupation and intensity of PS immunopositivity in the ONL (Fig2H). Exposure of PS at P21–22 was corroborated in unfixed cryosections of rd10 retina, using conjugated annexin V as a marker (Fig EV1B). Co-immunolabeling for rhodopsin and cone arrestin at P22 revealed that most PS exposure occurred in rod, rather than cone, photoreceptors (Fig2I–K), with a significantly greater proportion of rods showing PS labeling. These data demonstrate that mutation-bearing rods in the rd10 retina specifically present a phagocytic “eat-me” signal during the onset of rod degeneration.

### Microglial phagocytosis of rods during degeneration occurs concurrently but separately from rod apoptosis

**Figure EV2:**
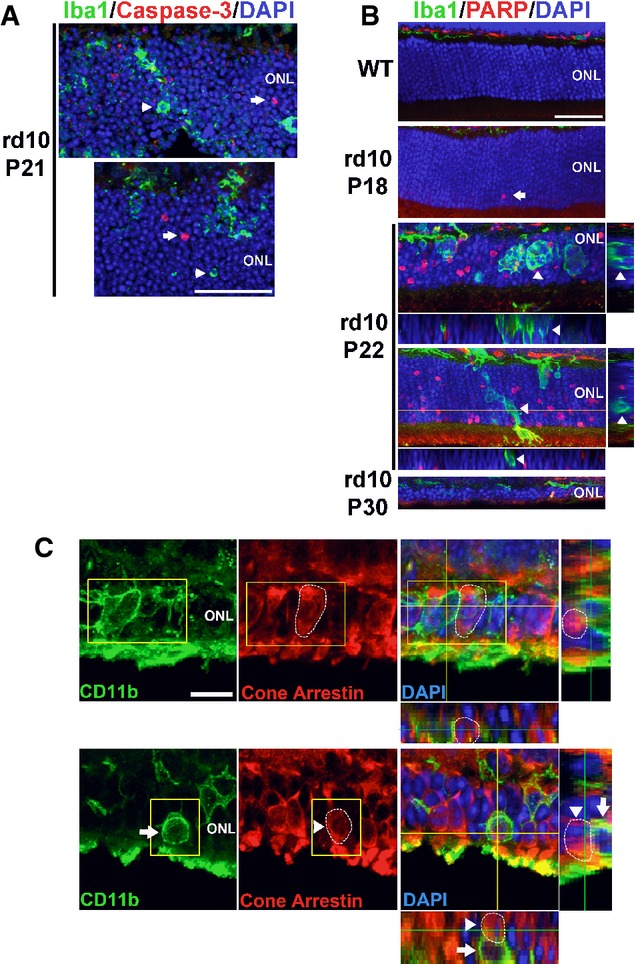
Infiltrating microglia phagocytose rods that are immunonegative for markers of early apoptosis and do not phagocytose cones during rod degeneration In P21 rd10 retina, ONL nuclei immunopositive for activated caspase-3 (red, arrows), an enzyme activated during early apoptosis, were found separately from phagocytized photoreceptor nuclei in microglial phagosomes (green, arrowheads). Scale bar, 40 μm.

**Figure 3:**
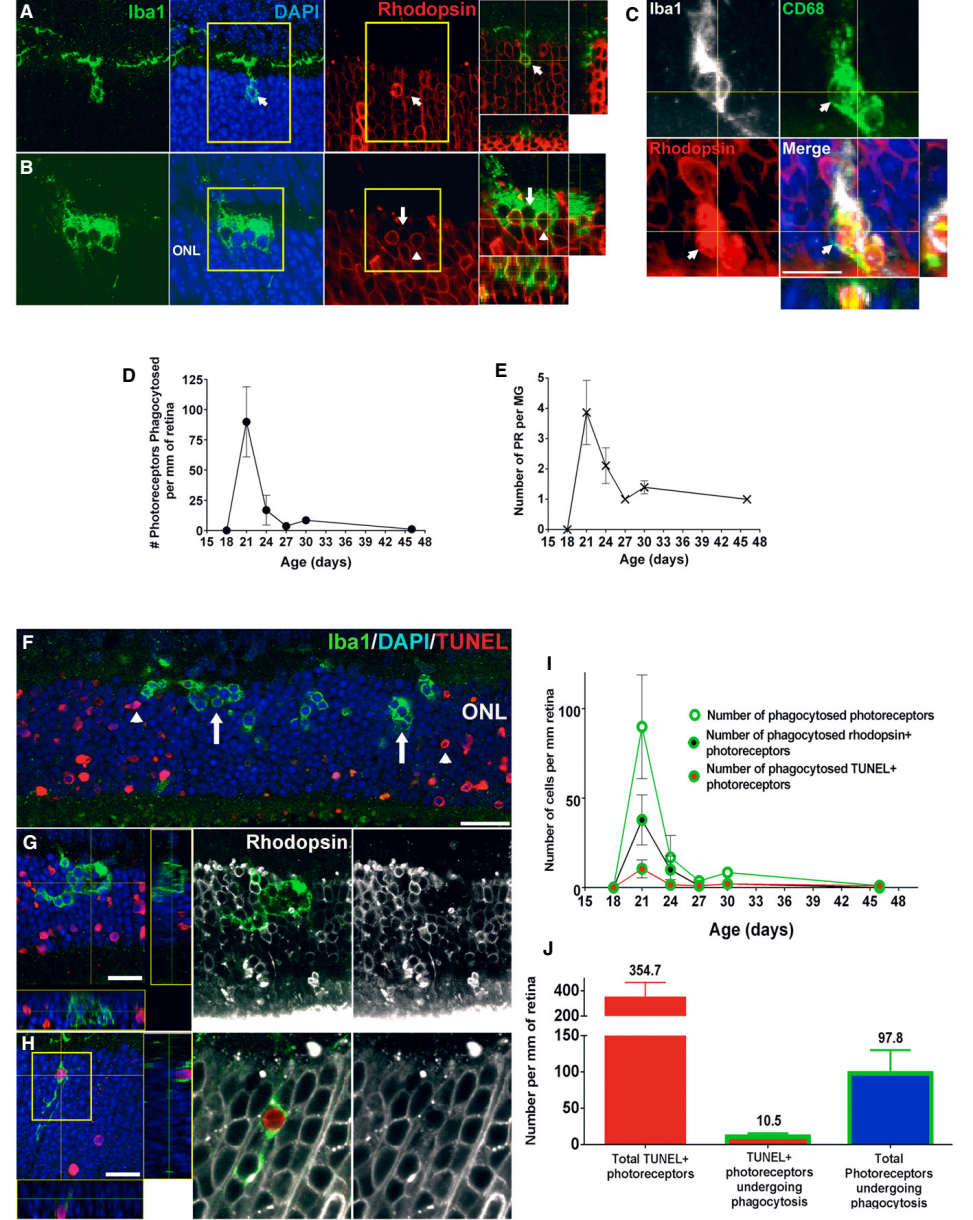
Infiltrating microglia phagocytose non-apoptotic photoreceptor rods during rod degeneration A–C Phagocytosis of rods by infiltrating microglia. (A) Representative example of a Iba1^+^ microglial process extending into the ONL with a phagosome at its terminal end. Each phagosome contained a photoreceptor nucleus (labeled with DAPI, arrow) that was identified as a rod photoreceptor by rhodopsin immunopositivity (superposition of Iba1^+^ phagosome with rhodopsin^+^ soma in orthogonal views). (B) Example of an amoeboid microglia in the ONL with multiple phagosomes containing both rhodopsin-positive (arrowhead) and rhodopsin-negative (arrow) nuclei. (C) Rhodopsin^+^ nuclei can be localized within CD68-positive phagosomes in infiltrating microglia, indicating phagocytic engulfment of rods. Scale bar, 10 μm.

**Figure 4:**
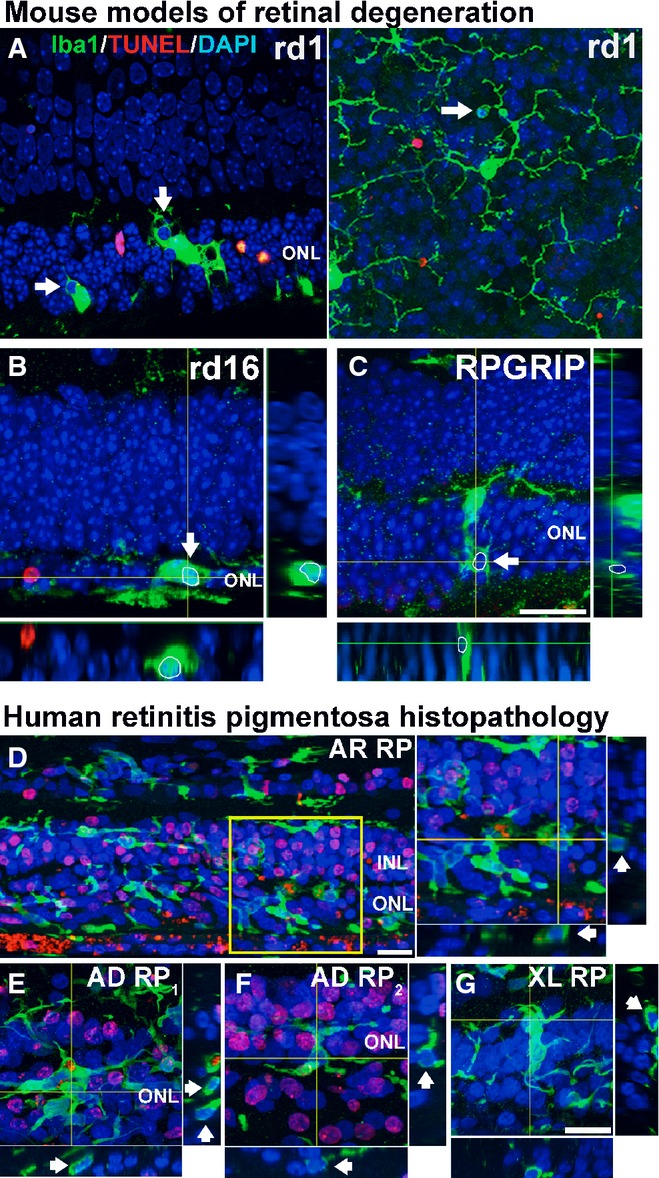
Microglial phagocytosis of photoreceptors in alternative mouse models of retinitis pigmentosa (RP) and in human histopathological specimens A–C Evidence of microglial phagocytosis of photoreceptors in other mouse models of RP. Histological analysis of a rd1 mouse retina (A) demonstrates microglial phagocytosis of photoreceptor nuclei in vibratome sections (left) and in flat-mounted retina (right). Phagocytosed nuclei were predominantly negative for TUNEL staining (arrows). Similar findings were found in the retinas of the rd16 mouse (loss-of-function mutation in the photoreceptor-expressed *CEP290* gene, 1 month old) (B) and the RPGRIP-deficient mouse (6 months old) (C). Scale bar, 20 μm.

The expression of phagocytic molecules in infiltrating microglia and the presence of “eat-me” signals on photoreceptor rods suggested that microglial phagocytosis of rods may contribute to degeneration. We performed rhodopsin and Iba1 immunolabeling across the period of rod degeneration and observed that infiltrating microglia contained DAPI^+^ nucleated cells that were immunopositive for rhodopsin (Fig3A and B), confirming microglial engulfment of rods. We also localized the engulfed rhodopsin-positive cells within CD68-positive microglial phagosomes (Fig3C), confirming that engulfment occurred in the context of microglial phagocytosis. Assessment of the level of microglial phagocytosis activity, quantified as the density of phagocytosed ONL nuclei (Fig3D) and the mean number of phagocytosed nuclei per infiltrating microglia (Fig3E), demonstrated a peak at P21 before decreasing thereafter. As microglial phagocytosis of neurons can involve either the late clearance of cells that have already undergone apoptosis (Ravichandran & Lorenz, 2007) or otherwise the primary phagocytosis of reversibly injured neurons (Brown & Neher, 2014), we performed TUNEL staining to discover whether apoptotic cells were the primary target of microglial phagocytosis. We found that while rod apoptosis (as indicated by TUNEL labeling) and phagocytosis (indicated by microglial engulfment) were present concurrently, they occurred for the most part discretely in separate photoreceptors (Fig3F and G). While many phagocytosed ONL nuclei were rhodopsin-positive, only a small minority of these (≈10%) were also TUNEL-positive (Fig3H and I). Even at the peak of rod degeneration at P21, when apoptotic TUNEL^+^ rods and microglia-engulfed rods were both highly prevalent in the ONL, only a small overlap was found between these two populations (Fig3J). The parallel but separate nature of these two processes was also supported by findings of predominant immunonegativity of phagocytosed photoreceptors for early markers for apoptosis, including activated caspase-3, as well as cleaved poly(ADP-ribose) polymerase (PARP) (Lazebnik *et al*, 1994), a substrate of activated caspase-3 (Fig EV2A and B). Microglial phagocytosis during the period examined appeared to be specific to rods; cone-arrestin-labeled cones were not detected within microglial phagosomes despite their close proximity to infiltrating microglia (three-dimensional colocalization analysis recorded 0/12 arrestin^+^ cones engulfed by microglia, and 0/19 microglia containing arrestin immunopositivity in any intracellular phagosomes, *n* = 3 animals) (Fig EV2C). Histological analyses of mouse models of RP involving an alternative mutation in Pde6b (rd1), and mutations in other photoreceptor genes (rd16 and RPGRIP^−/−^), demonstrated similar evidence of microglial phagocytosis of TUNEL–photoreceptors (Fig4A–C), as did histopathological specimens of human RP, including autosomal recessive, autosomal dominant, and X-linked forms (Fig4D–G), indicating microglial phagocytosis of stressed rods as a generalized mechanism underlying RP.

Immunohistochemistry for cleaved poly(ADP-ribose) polymerase (PARP), a substrate of activated caspase-3, shows the absence of immunopositive ONL nuclei in adult wild-type (WT) mouse. In the rd10 retina, immunopositive ONL nuclei were rare at P18, prevalent at P22, and decreased at P30, as expected from the progression of rod degeneration. At P22, ONL nuclei phagocytosed by microglia (arrows in insets) are predominantly immunonegative for cleaved PARP (arrowheads in orthogonal views). Scale bar, 40 μm.

Photoreceptor cones are not phagocytosed by microglia during rod degeneration. (Upper panels) At P21–23, although infiltrating microglia in the ONL (CD11b, green) contain phagocytosed nuclei (DAPI, blue), none of these were found to be associated with cone arrestin immunopositivity (red), despite the close proximity of arrestin-positive cone somata (highlighted by circled area) to infiltrating microglia. (Lower panels) Example of a CD11b-positive microglial cell in the ONL juxtaposed closely to an arrestin-positive soma (highlighted by circled area). Analysis of orthogonal views of the confocal image stack demonstrates the absence of cone phagocytosis by microglia. Scale bar, 10 μm.

D, E The peak of microglial phagocytic activity occurred around P21 and declined subsequently, as measured by the number of rods phagocytosed (D) and the number of photoreceptors phagocytosed per microglial cell (E) (*n* = 3 animals at each time point).

F–J Infiltrating microglia phagocytose TUNEL-negative rods in the ONL. (F) Although the peaks of TUNEL positivity and microglial phagocytosis occurred concurrently, the majority of rod photoreceptors undergoing microglial phagocytosis were non-apoptotic (arrows), while many apoptotic photoreceptors were not phagocytosed by microglia (arrowheads); representative example of a P21 retina demonstrates non-overlapping patterns of TUNEL positivity (red) and microglial phagocytosis (Iba1, green). (G) Confocal analysis of a representative amoeboid microglial cell demonstrates that while the photoreceptor nuclei in phagosomes were rhodopsin-positive (*white*), they were mostly TUNEL-negative. Only a small fraction of microglial phagosomes contained TUNEL^+^, rhodopsin^+^ rods (example shown in H). Scale bars, 20 μm. (I) Quantitative analysis of photoreceptor nuclei within microglial phagosomes according to rhodopsin (black symbols), and TUNEL labeling (red symbols) at different times during rod degeneration. (J) Quantitative analysis of all photoreceptors undergoing either apoptosis or phagocytosis at P21–23 demonstrates that TUNEL^+^ apoptotic photoreceptors and phagocytosed photoreceptors consist of two distinct populations, indicating separate but parallel mechanisms of rod degeneration. Quantitative analyses involved retinal sections from *n* = 3 animals at each age. Data information: Data points in (D, E, I) and column heights in (J) indicate mean, error bars indicate ± SEM.

D–G Evidence of microglial phagocytosis of photoreceptors in other human histopathological specimens of RP. (D) Retinal section from a 30-year-old male donor with autosomal recessive RP (AR RP) showing extensive microglial infiltration of the ONL; expanded inset (right) shows multiple photoreceptor nuclei in phagosomes that were predominantly negative for TUNEL staining. (E, F) Retinal sections from two separate donors with autosomal dominant RP (AD RP_1_ = 68-year-old man, T17M rhodopsin mutation, AD RP_1_ = 50-year-old woman, Q-64-ter rhodopsin mutation) showing similar evidence of microglial phagocytosis. (G) Retinal section from a 46-year-old male donor with X-linked RP. Arrowheads indicate phagocytosed photoreceptor nuclei. Scale bars, 20 μm.

### Dynamic interactions underlying the clearance of rod photoreceptors by microglial phagocytosis

**Figure 5:**
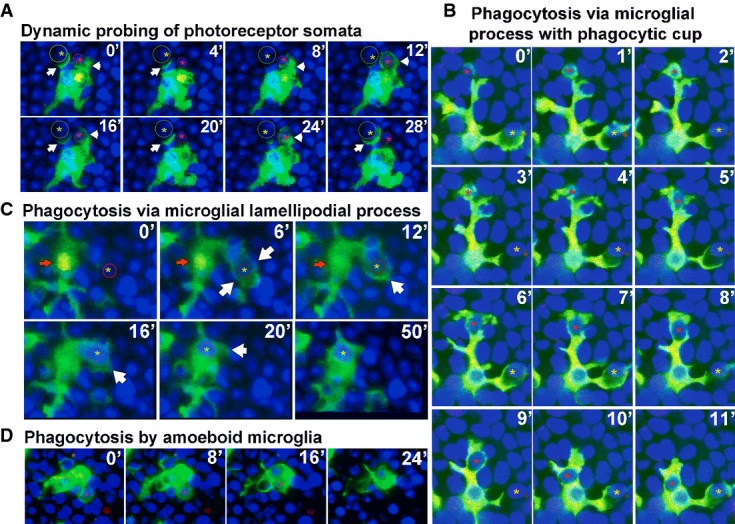
Dynamic interactions between photoreceptors and infiltrating microglia underlying phagocytosis during photoreceptor degeneration Time-lapse confocal imaging (at a rate of one image stack/min) was used to document dynamic behavior of ONL microglia in rd10 retinal explants at ages P21–24.
Infiltrating microglia established rapid and transient physical contacts with nearby photoreceptor somata (red and yellow circles) via their processes; these processes often terminated in a “cup”-like structure (arrow) that upon soma contact proceeded to extend across the entire soma (arrowhead, and red circle and *) to engulf it completely. The majority of microglia–photoreceptor contacts were transient with the microglial processes dynamically contacting and releasing photoreceptor somata in repetitive cycles (e.g. yellow circle and *) (period ≅10–15 min).

To further understand how infiltrating microglia interact with photoreceptors in the ONL during rod degeneration, we conducted live *ex vivo* imaging of microglial behavior in acutely isolated retinal explants from CX3CR1^GFP/+^/rd10 mice. Photoreceptor nuclei were vitally labeled with Hoechst 33342 stain, and rods undergoing late apoptosis were co-labeled with propidium iodide (PI), which was excluded by viable rods. We observed that infiltrating ONL microglia demonstrated constitutive dynamism in their processes that made repeated focal contact with photoreceptor somata. These dynamic processes often terminated in a specialized cup-like structure, which extended around photoreceptor somata to variable extents (Fig5A). These “probing” contacts were typically transient and repetitive, with the steps of: (i) process extension, (ii) soma contact, (iii) partial envelopment of soma, and (iv) release and process retraction, occurring in cycles of ≈10–15 min of duration (Movie EV1). In a subset of such contacts, the extension of the phagocytic “cup” progressed to entirely engulf the photoreceptor soma, sequestering the cell in an intracellular phagosome, which was subsequently translocated intracellularly toward the microglial cell body (Fig5B; Movie EV2). These stages of: (i) soma contact, (ii) complete soma engulfment, and (iii) phagosome translocation occurred over a period of ≈10–15 min. Phagocytosis was also observed to occur via engulfment by lamellipodial microglial processes without a defined phagocytic cup (Fig5C; Movie EV3) or at the soma of amoeboid microglial cells lacking extended processes (Fig5D; Movie EV4). While infiltrating microglia were observed to “probe” both PI^+^ and PI^−^ cells via their processes, overt phagocytosis selectively involved PI^−^ cells (out of 37 phagocytic events scored, 36/37 involved PI^−^ somata vs. 1/37 involving a PI^+^ soma, *n* = 8 recordings in eight animals), supporting the earlier observations that microglial phagocytosis primarily involves viable photoreceptors. Following phagocytosis, Hoechst^+^, PI^−^ rods retained within a phagosome (for up to 30 min) were observed to develop PI nuclear staining, followed by a gradual (≈10 min) disappearance of nuclear staining (Fig5C), indicating membrane permeabilization and cellular breakdown within the phagosome. We estimate that the entire phagocytosis process of a single rod, from initial microglial contact to eventual intracellular breakdown, occurs over the time-scale of ≈1 h. We note that spatial range in the ONL over which a single microglial cell can phagocytose cells is augmented by the microglial cell’s ability to extend long processes dynamically, interact simultaneously with multiple photoreceptor somata, and migrate through the ONL (Movie EV5).

Example in which the engulfment of photoreceptor somata by microglial phagocytic cup was followed up by actual phagocytosis in which the engulfed cell (red *) is translocated intracellularly within the microglial cell toward the cell body. Phagocytosis of soma occurred simultaneously with “probing” of other somata by processes of the same microglial cell (yellow *).

Phagocytosis of photoreceptor somata occurred via flattened microglial lamellipodial microglial processes (arrow) that extended across somata (yellow *) to engulf them. Nuclei of phagocytosed photoreceptors within microglial phagosomes occasionally developed staining for propidium iodide (red arrow); these subsequently faded and disappeared over ≅10 min, possibly representing intraphagosomal breakdown.

Amoeboid infiltrating microglia lacking extended processes were also observed to phagocytose photoreceptors via phagocytic “cups” formed at their cell body (red *).

### Genetic ablation of infiltrating retinal microglia ameliorates rod photoreceptor degeneration

**Figure 6:**
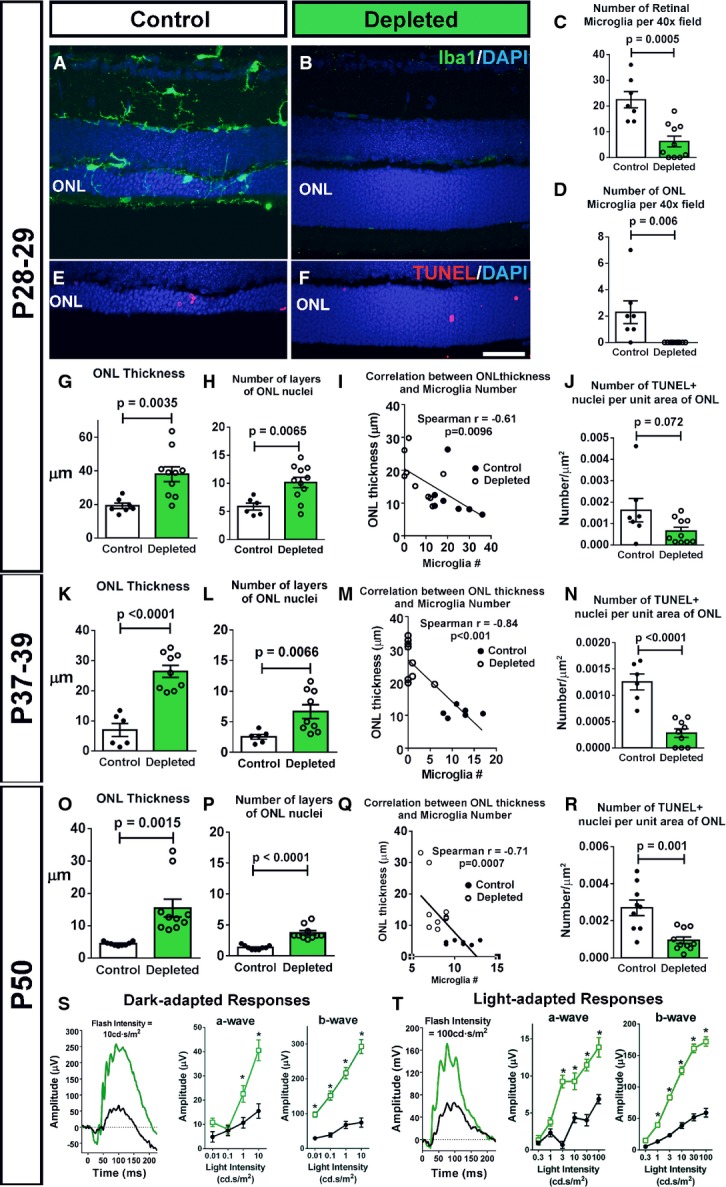
Genetic ablation of microglia in the retina ameliorates photoreceptor degeneration in rd10 mice Retinal microglia were depleted in rd10/CreDTA mice by the oral administration of tamoxifen (in corn oil) to activate microglia-specific Cre-mediated recombination and diphtheria toxin expression; control CreDTA littermates were administered corn oil without tamoxifen.
A–D Depletion of retinal microglia in the rd10 retina. Representative retinal section from a P28 control animal demonstrates Iba1^+^ microglia in the retina, including those infiltrating the ONL (A), while a tamoxifen-administered littermate (B) was substantially depleted of retinal microglia. Scale bar, 40 μm. Microglial cell counts in the entire retina (C) and in the ONL only (D) confirmed efficient depletion of infiltrating microglia following tamoxifen administration (*n* = 8 control and 11 depleted animals from four litters, two-sided unpaired *t*-test).

To examine the contribution of infiltrating microglia to overall rod degeneration in rd10 mice, we evaluated the effects of depleting microglia from the retina at the time of degeneration. We employed a transgenic mouse model (CX3CR1^CreER^ × Rosa26-flox-STOP-flox –DTA mice) in which tamoxifen-inducible Cre recombinase (CreER) is specifically expressed under the control of the endogenous CX3CR1 promoter, allowing CX3CR1^+^ retinal microglia to express diphtheria toxin and be specifically ablated upon tamoxifen administration (Parkhurst *et al*, 2013). We crossed these mice into the rd10 background to generate rd10/CreDTA mice and depleted the retina of microglia by tamoxifen administration at P21–23. Analysis at P28–29 demonstrated efficient depletion of retinal microglia, including infiltrating ONL microglia (Fig6A–D). Microglial depletion during rod degeneration resulted in a significantly greater preservation of ONL thickness in depleted animals relative to undepleted control littermates (Fig6G and H). The degree of ONL preservation correlated significantly with the extent of microglial depletion (fewer residual microglia correlating with increased ONL thickness) (Fig6I), underscoring the relationship between infiltrating microglia and ONL degeneration. Significant morphological rescue effects persisted when tamoxifen-induced microglial depletion was maintained until P37–39 (Fig6K–M) and P50 (Fig6O–Q), with greater levels of depletion again corresponding to greater extents of morphological rescue. Interestingly, the density of TUNEL^+^ nuclei in the ONL was also reduced by microglial depletion at all three time points examined (Fig6J, N and R). Functional rescue of degeneration was evident by electroretinography (ERG) performed at P50; both dark- and light-adapted responses demonstrated markedly increased a- and b-wave amplitudes at multiple flash intensity levels (Fig6S and T).

E–J Effect of microglial depletion on retinal degeneration at P28–29. ONL atrophy and thinning in control animals (E) was significantly more advanced relative to microglia-depleted littermates (F). Scale bar, 40 μm. Quantification of mean ONL thickness (G) and mean number of layers of ONL nuclei (H) at P28–29 demonstrate significantly greater ONL preservation in depleted retinas; the degree of ONL preservation correlated with the extent of microglia depletion (I). (J) The mean density of TUNEL^+^ nuclei in the ONL was not significantly decreased in depleted vs. control animals (*n* = 8 control and 11 depleted animals from four litters, two-sided unpaired *t*-test).

K–N Continuation of microglial depletion until P37–39 resulted in the persistence of morphological rescue (K–M), with a significant reduction in TUNEL^+^ nuclei density (N) (*n* = 6 control and nine depleted animals from two litters, two-sided unpaired *t*-test).

O–R Similar rescue effects as in (K–N) remained apparent when depletion was sustained until P50 (O–R), a time when rod degeneration in the rd10 model is relatively complete (*n* = 9 control and 10 depleted animals from three litters, two-sided unpaired *t*-test).

S, T Functional rescue of photoreceptors was evident following microglial depletion until P50 in significantly increased dark- (S) and light-adapted (T) responses in ERG testing in depleted animals (green lines) relative to control animals (black lines), in both a- and b-wave amplitudes across multiple flash intensities (*n* = 9 control and nine depleted animals, *P < 0.05 in one-way ANOVA with Sidak’s multiple comparison test). Data information: Column heights (in C, D, G, H, J, K, L, N, O, P, R) and data points (in S, T) indicate mean, error bars indicate ± SEM.

### Inhibition of microglial phagocytosis ameliorates rod photoreceptor degeneration

**Figure 7:**
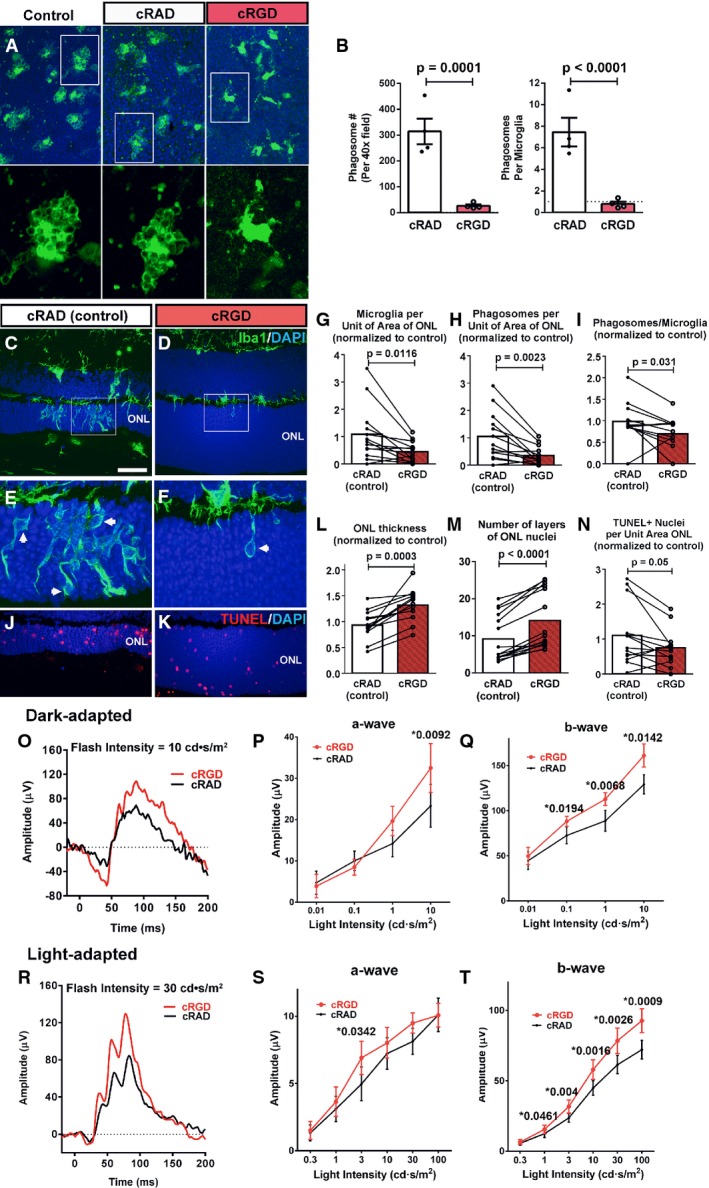
Inhibition of microglial phagocytosis ameliorates photoreceptor degeneration in rd10 mice A, B Effect of phagocytosis inhibition on retinal microglia *ex vivo*. Retinal explants acutely isolated from P21–23 rd10/CX3CR1^GFP/+^ mice were incubated in Ringer’s solution (control), or Ringer’s solution containing either the vitronectin receptor inhibitor cRGD peptide or its inactive analog cRAD (400 μM) for 1 h. GFP-labeled (green) ONL microglia in explants in cRGD transitioned from amoeboid morphologies containing multiple phagosomes to more ramified morphologies with fewer phagosomes. No morphological changes were detected in microglia incubated in cRAD (insets show morphologies at higher magnification). Scale bar, 40 μm. Quantifications of total number of phagosomes per 40× field (left) and the mean number of phagosomes per microglia (right) demonstrate significant reductions in phagocytic activity of cRGC, relative to cRAD, exposure (*n* = 8 imaging fields from in each condition).

To explore the specific contribution of microglial phagocytosis on the progression of rod degeneration, we employed cRGD, a specific inhibitor of the vitronectin phagocytosis receptor (Aumailley *et al*, 1991; Neher *et al*, 2011), to inhibit microglial phagocytosis. We found that incubation of retinal explants from P21–23 rd10 mice in Ringer’s solution containing cRGD (400 μM, 1–4 h) resulted in a marked reduction in the number of phagosomes and more ramified morphologies in infiltrating ONL microglia relative to untreated control explants or explants incubated in cRAD, the inactive analog of cRGD (Fig7A and B), demonstrating effective phagocytosis inhibition. To investigate the effects of phagocytosis inhibition *in vivo*, we performed intravitreal injections of cRGD into one eye of P20 rd10 mice, with cRAD injected in the control contralateral eye. Morphological analyses at P23 revealed that cRGD-injected eyes had significantly fewer infiltrating microglia and microglial phagosomes in the ONL (Fig7C–H), as well as a decreased number of phagosomes per microglia (Fig7I). cRGD-injected eyes also demonstrated a significantly greater ONL preservation relative to contralateral cRAD-injected eyes (Fig7J–M). The density of TUNEL^+^ apoptotic nuclei in the ONL was also lower in cRGD-injected eyes, a difference that bordered on statistical significance (Fig7N).

C–N Structural effects of *in vivo* inhibition of microglial phagocytosis. P20 rd10 mice were injected intravitreally in one eye with the phagocytosis inhibitor cRGD, and in the control contralateral eye with the inactive analogue, cRAD. At P23, prominent microglia infiltration in the ONL observed in control-injected eyes (C) was decreased in the contralateral cRGD-injected eyes (D). ONL microglia in control eyes demonstrated more numerous phagosomes (inset, arrows) (E) compared with cRGD-injected eyes (F). Pairwise comparisons of control- vs. cRGD-injected eyes demonstrated that phagocytosis inhibition significantly reduced the densities of infiltrating microglia (G) and microglial phagosomes (H), and mean phagosome number per microglia (I). ONL atrophy in control eyes (J) was more advanced compared to cRGD-injected eyes (K), with significantly greater mean ONL thickness (L), and ONL nuclear layers (M) in cRGD-injected eyes. Mean density of TUNEL^+^ ONL nuclei was lower, with marginal significance (N) (*n* = 13 animals; measurements in cRGD-injected eyes normalized to contralateral control eyes, two-sided paired *t*-test). Scale bar, 40 μm.

O–T Functional effects of *in vivo* inhibition of microglial phagocytosis. Representative dark-adapted ERG recordings at P26 (following intravitreal injections at P20 and P23) in a cRGD-injected eye (red) relative to the contralateral control eye (black) (O) showing that mean a- (P) and b-wave (Q) amplitudes were significantly greater in the cRGD-injected vs. control eyes at higher flash intensities. Representative light-adapted responses (R) illustrating that while mean light-adapted a-wave amplitudes (S) were slightly but not significantly greater in cRGD-injected eyes, mean b-wave amplitudes (T) were significantly greater at higher flash intensities (*n* = 13 animals, two-sided paired *t*-test, *P < 0.05, with exact values alongside). Data information: Column heights (in B, G, H, I, L, M, N) and data points (in P, Q, S, T) indicate mean, error bars indicate ± SEM.

To evaluate the functional effects of phagocytosis inhibition, cRGD injections were performed in rd10 mice at P20 and P23, and *in vivo* electroretinogram (ERG) recordings obtained at P26. Dark-adapted responses were greater in cRGD-injected relative to contralateral cRAD-injected eyes, with significantly greater mean a-wave and b-wave amplitudes at higher flash intensities (Fig7O–Q). In cRGD-injected eyes, light-adapted a-wave amplitudes were slightly but not significantly increased, while b-wave amplitudes were significantly greater than in control eyes (Fig7R–T). These data corroborated our morphological analyses and indicate that specific inhibition of microglial phagocytosis ameliorated functional degeneration in the rd10 mouse.

### Infiltrating microglia in the ONL upregulate IL-1β expression potentiating photoreceptor apoptosis

**Figure 8:**
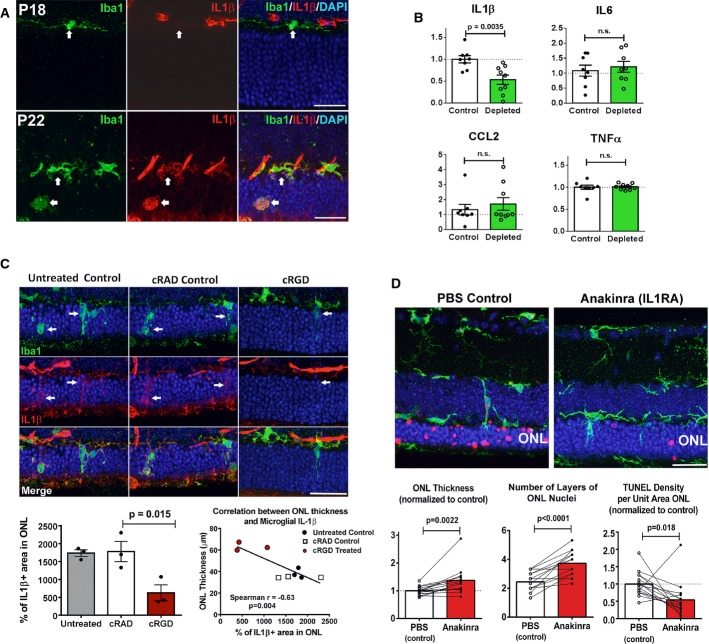
Upregulation of IL-1β expression in infiltrating microglia potentiates photoreceptor apoptosis Microglia infiltrating the rd10 retina upregulate IL-1β expression during degeneration. Iba1^+^ microglia (green) were immunonegative for IL-1β (red) at P18 but become prominently IL-1β immunopositive at P22 (arrows). Scale bars, 25 μm.

Our observations above indicate that microglial depletion and microglial phagocytosis inhibition, in addition to diminishing microglial phagocytic clearance of stressed but living rods, decreased the density of TUNEL^+^ photoreceptors. This suggested that infiltrating microglia may produce secreted factors such as proinflammatory cytokines that can potentiate photoreceptor apoptosis. In particular, microglial production of IL-1β has been associated with neuronal (Sivakumar *et al*, 2011) and capillary endothelial cell apoptosis (Rivera *et al*, 2013) in the retina, and cognitive decline in the brain (Cho *et al*, 2015). We found in immunohistochemical studies that while ramified non-infiltrating microglia at P18 are immunonegative for IL-1β, infiltrating microglia in the ONL at P22 develop prominent immunopositivity, indicating microglial IL-1β upregulation in the rd10 retina (Fig8A). Consistent with this, microglial depletion in the rd10 retina significantly lowered retinal levels of IL-1β as assessed by ELISA, but not IL6, CCL2, or TNFα (Fig8B). Similarly, inhibition of microglial phagocytosis by intravitreal injection of cRGD effectively decreased IL-1β immunopositivity in infiltrating microglia, with no significant changes seen in cRAD controls (Fig8C). The degree of ONL preservation also correlated negatively with the extent of ONL IL-1β immunopositivity in experimental animals, suggesting a causal association. To directly assess the ability of microglial-derived IL-1β to contribute to photoreceptor degeneration via pro-apoptotic mechanisms, we inhibited IL-1β signaling during retinal degeneration by intravitreal injections of anakinra, a recombinant IL-1 receptor antagonist (IL-1-RA) at the start of rod degeneration. Anakinra-injected eyes, relative to contralateral, PBS-injected control eyes, demonstrated a greater ONL preservation and a lower density of TUNEL^+^ nuclei in the ONL (Fig8D). These data indicated that infiltrating microglia, in addition to phagocytosing rods, contribute additionally to rod degeneration by potentiating apoptosis through IL-1β upregulation.

Depletion of infiltrating microglia in the rd10 retina decreases IL-1β levels. Cytokine levels in rd10/CreDTA mouse retinas were assayed following tamoxifen-induced microglial depletion (from P21 to P37–50, green bars; *n* = 9 animals) and compared with their untreated littermate controls (white bars, *n* = 8 animals; values normalized to control animals in the same litter). IL-1β protein levels were significantly lowered following microglial depletion, but IL-6, CCL2, or TNFα were not significantly changed.

Inhibition of microglial phagocytosis results in decreased microglial IL-1β expression. Eyes of rd10 animals were treated with intravitreal injections of cRGD at P20, and analyzed at P23 by immunohistochemistry for Iba1 (green) and IL-1β (red), and compared to uninjected eyes and with eyes injected with inactive analog, cRAD (*n* = 3 eyes in each category). Immunopositivity was high in infiltrating microglia (indicated by arrows) for untreated and cRAD-injected control eyes and significantly reduced in microglia in cRGD-injected eyes (upper panel), as demonstrated by the quantification of IL-1β expression (area of immunopositivity as a fraction of the ONL) (lower left panel). ONL thickness correlated negatively with the extent of IL-1β immunopositivity (lower right panel). Scale bar, 25 μm.

IL-1R signaling during rod degeneration was inhibited by intravitreal injections of recombinant IL-1 receptor antagonist, anakinra (from P20–22 to P26–27) in one eye of each rd10 animal, while the contralateral eye was injected with PBS as a control. Upper panels show representative retinal sections from control PBS-injected and IL-1RA-injected eyes from the same animal. ONL atrophy in control eyes was more advanced compared to IL-1RA-injected eyes, with greater mean ONL thickness and lower mean density of TUNEL^+^ ONL nuclei in IL-1RA-injected eyes (lower panels) (*n* = 15 animals; measurements in IL-1RA-injected eyes normalized to contralateral control eyes, paired *t*-test). Scale bar, 25 μm. Data information: Column heights (in B, C, D) indicate mean, error bars indicate ± SEM.

## Discussion

Our results demonstrate that retinal microglia are prominently involved in inherited retinal degeneration and that microglial phagocytosis of living rods is a mechanism contributing to overall rod demise. At the onset of rod degeneration in the rd10 retina, microglia infiltrate the ONL, a zone normally exclusionary of microglia (Roque *et al*, 1996) and become activated. Although the signals attracting and activating microglia are undefined, they likely involve “find-me” signals (Ravichandran, 2011) such as nucleotides (ATP, UTP) and fractalkine, which can be released extracellularly from mutation-bearing photoreceptors (Notomi *et al*, 2011). These can also induce infiltrating microglia to adopt an activated, phagocytic phenotype (Inoue *et al*, 2009) incorporating phagosome formation, upregulation of phagocytic molecules, and secretion of phagocytic bridging molecules (Miksa *et al*, 2007). Within the ONL, infiltrating microglia come into direct contact with stressed but viable rods that expose PS on their surfaces, marking them as phagocytic targets. PS exposure in neurons results from inhibition of PS translocase (Levano *et al*, 2012) which can be induced by intracellular calcium dysregulation (Suzuki *et al*, 2013) or oxidative stress (Tyurina *et al*, 2007; Vlachantoni *et al*, 2011) that occur in mutation-bearing rods (Sancho-Pelluz *et al*, 2008). Neher and colleagues have demonstrated that PS exposure in neurons under neuroinflammatory conditions does not represent an irreversible commitment to apoptosis, but rather a presentation of an “eat-me” signal by stressed but living neurons that, left unphagocytized, can continue to be viable (Neher *et al*, 2011). Similar findings are found in growth-factor-deprived neurons (Kim *et al*, 2010) and in transformed cells *in vitro* (Hammill *et al*, 1999; Geske *et al*, 2001; Segawa *et al*, 2011). PS exposure occurred primarily in rods during rod degeneration; a minority of cones showing PS exposure were spared from phagocytosis, indicating that additional regulatory signals (“eat-me” and “don’t eat-me signals) (Brown & Neher, 2012) may be involved in conferring the rod specificity to microglial phagocytosis.

Our live-imaging observations revealed for the first time the dynamic nature of microglia–photoreceptor interactions during photoreceptor degeneration. We observed that infiltrating microglia selectively target a few nearby photoreceptor somata by making contact via extending processes that end in phagocytic cups. These cups make repeated partial and abortive engulfments of selected photoreceptor somata before eventually progressing to full engulfment and phagocytosis. These features suggest that microglia can detect cell-surface cues on stressed photoreceptors that preferentially “prime” them for phagocytosis and are engaged in repeated assessment of their targets prior to overt phagocytosis. It is likely that microglia via these repeated contacts actively modify photoreceptor morphology, inducing photoreceptor axon and dendrite retraction. Activated microglia *in vitro* have been shown to induce neurite and axon retraction, either via secreted factors (Munch *et al*, 2003) or via direct dynamic microglial contact (Horn *et al*, 2008) mediated through the RhoA/Rho-associated kinase (ROCK) pathway (Borrajo *et al*, 2014). Direct visualization of changes of photoreceptor morphology induced by infiltrating microglia using live-cell imaging will be instructive in further understanding microglia–photoreceptor interactions.

While microglial infiltration in the rd10 retina first originates from the migration and process extension of inner retinal microglia, systemically recruited monocytes may also enter into the outer retina. A previous study has reported that rd10 mice lacking CCR2 (rd10, CCR2^−/−^), the chemokine receptor implicated in monocyte recruitment, demonstrated modestly reduced numbers of retinal F4/80^+^ cells (≈10% decrease) and slightly less rod degeneration relative to rd10, CCR2^+/+^ mice, indicating a partial contribution from systemically recruited monocytes to overall neurodegeneration (Guo *et al*, 2012). However, our observations from histological and live-imaging studies in retinas of rd10, CX3CR1^+/GFP^, CCR2^+/RFP^ mice (in which endogenous microglia and recruited monocytes can be distinguished by RFP expression) (Mizutani *et al*, 2012) indicate that recruited monocytes are not likely to significantly contribute to phagocytic clearance of photoreceptors as they: (i) were largely located in the subretinal space, rather than the ONL, (ii) lacked processes to engulf photoreceptors and do not contain intracellular phagosomes that indicate recent phagocytic behavior, and (iii) did not display dynamic phagocytic behavior observed in endogenous infiltrating microglia (FigEV3; Movie EV6). As such, resident microglia, rather than recruited monocytes, are likely to be primarily responsible for phagocytic clearance of stressed rods in retinal degeneration.

**Figure EV3:**
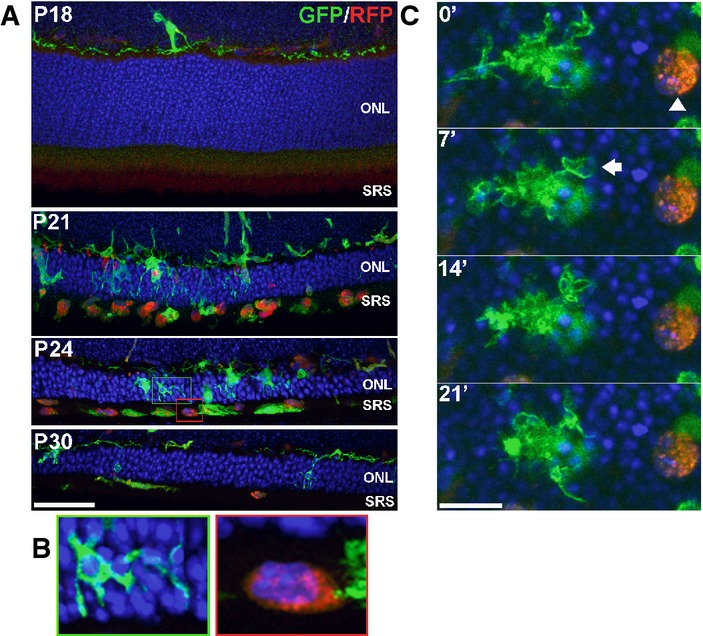
Infiltrating retinal microglia, endogenous to the retina, primarily mediate photoreceptor phagocytosis, with little or no participation of systemically recruited monocytes Retinas from transgenic CX3CR1^+/^^GFP^, CCR2^+/^^RFP^ mice crossed into the rd10 background were examined at time points before and during rod degeneration. At P18, prior to degeneration, the outer nuclear layer (ONL) and subretinal space (SRS) were relatively devoid of infiltrating cells. At P21, numerous GFP^+^, RFP^−^ retinal microglia infiltrate the ONL and engulf photoreceptors. Concurrently, a number of RFP^+^ monocytes with rounded morphologies appeared predominantly in the SRS, with only a few cells within the ONL. At P24 and P30, RFP^+^ monocytes remained concentrated in the SRS and declined in number with time. Scale bar, 40 μm.

Morphological features of GFP^+^, RFP^−^ retinal microglia and RFP^+^ monocytes differ significantly from each other. Infiltrating microglia (from green inset from P24 in A) demonstrated multiple processes contacting and engulfing photoreceptors in phagosomes, while RFP^+^ monocytes (red inset) lacked processes, had limited contact with photoreceptors, and lacked evidence of intracellular phagosomes.

Dynamic behavior also differed significantly between the two cell types as observed in a P24 retina explant by live confocal imaging: a GFP^+^, RFP^−^ retinal microglial cell (left) demonstrated typical dynamic process movement and engulfment (arrow), while RFP^+^ monocytes (arrowhead) were relatively stationary and lacked dynamic process behavior. Scale bar, 10 μm.

As found in previous studies of primary microglial phagocytosis (Brown & Neher, 2014), we discovered that microglia in the rd10 retina are primarily involved in the phagocytosis of living non-apoptotic rods, rather than merely engaged in the clearance of dead and apoptotic cells. This was evidenced by histological data demonstrating that phagocytosed rhodopsin^+^ rods were predominantly negative for both early (activated caspase-3 and cleaved PARP) and late (TUNEL) markers of apoptosis, and by live-imaging observations of microglia phagocytosing predominantly PI-negative, rather than PI-positive, cells. In addition, if microglial phagocytosis served solely to clear apoptotic cells, then microglial depletion and microglial phagocytosis inhibition will not be expected to result in the functional and structural rescue of rod degeneration observed here. Although rod apoptosis and phagocytosis occur concurrently, it is difficult to estimate their relative contributions to rod demise; while we had documented a greater apparent prevalence of TUNEL^+^ rods vs. phagocytosed rods at individual time points, the duration of rod phagocytosis by microglia (from initial contact to phagosomal breakdown) is relatively short (estimated to be ≈1 h) relative to the greater duration of TUNEL positivity in an apoptotic rod, which may lead to an underestimation of the contribution of microglial phagocytosis in rod degeneration.

As microglia have been hypothesized to play key homeostatic functions in the healthy CNS (Katsumoto *et al*, 2014), a question arises as to whether photoreceptor survival may itself be influenced by the absence of microglia *per se*. We have previously found using a similar genetic system that microglial depletion in the undiseased adult mouse brain had little influence on overall neuronal and synaptic density, on neuronal apoptosis, or the integrity of the blood–brain barrier (Parkhurst *et al*, 2013). In the retina, we have similarly found that prolonged depletion of retinal microglia of up to 30 days in an undiseased adult mouse retina does not negatively influence retinal lamination and structure, or induce photoreceptor apoptosis and atrophy (*unpublished data*). Based on these findings, it is likely that retinal microglia in the mature healthy retina, over the relatively short time-scale of our experiments here, are dispensable for photoreceptor survival. On the other hand, in pathological situations, such as in the degenerating rd10 retina, retinal microglia can transition to an activated, pro-phagocytic, pro-apoptotic state that is significantly deleterious to photoreceptor survival.

We had observed interestingly that while microglial depletion and microglial phagocytosis inhibition exerted rescue effects by decreasing microglial clearance of living rods, these measures also resulted in a significantly decreased density of TUNEL^+^ photoreceptors. This raised the possibility that activated microglia infiltrating the ONL can further potentiate rod apoptosis that is triggered by the Pde6b mutation. We observed here that infiltrating phagocytic microglia indeed demonstrate markers of activation and express increased IL-1β. While microglial phagocytosis of apoptotic cells can downregulate inflammatory responses in some contexts (Magnus *et al*, 2001; De Simone *et al*, 2003), phagocytosis of photoreceptor proteins has been found to conversely increase microglial activation *in vitro* and elevate expression of pro-inflammatory cytokines, such as IL-1β and TNFα, and chemotactic cytokines, such as CCL2 (Kohno *et al*, 2013). Consistent with this, we found that inhibiting microglial phagocytosis *in vivo* with the vitronectin receptor antagonist, cRGD, increased microglial ramification, decreased microglial IL-1β immunopositivity, and diminished microglial infiltration. *In vivo* inhibition of microglial-mediated IL-1 β signaling using recombinant IL-1RA was indeed effective in decreasing photoreceptor TUNEL staining, demonstrating the pro-apoptotic effect of activated microglia, which is in turn related to microglial phagocytosis.

**Figure 9:**
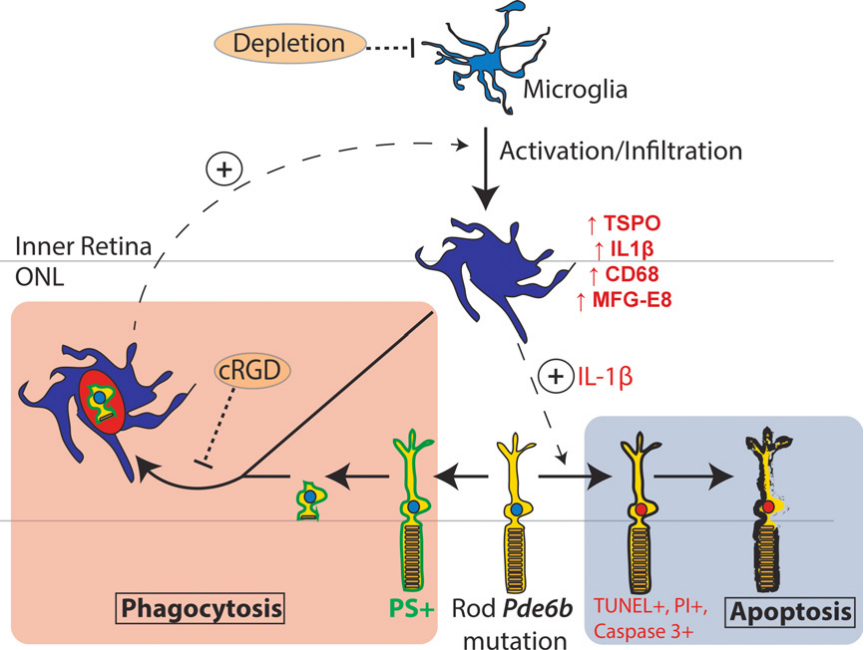
Schematic illustrating the non-cell-autonomous contributions of infiltrating retinal microglia to rod demise in inherited retinal degenerations In the rd10 mouse, rod photoreceptors bearing a mutation in the Pde6b protein experience cellular stress. A subset of rods undergoes apoptotic cell death (blue box) which is marked by the development of TUNEL, PI, and activated caspase-3 staining. Ramified microglia in the inner retina, sensing photoreceptor stress via unknown signals, infiltrate the outer nuclear layer (ONL) shortly after P18, expressing markers of activation (e.g., TSPO), pro-inflammatory cytokines (e.g., IL-1β), and phagocytic molecules (e.g., CD68 and MFG-E8). A subset of stressed but viable rods increases the presentation of the cell-surface “eat-me signal” phosphatidylserine (PS, green), inducing direct cell–cell contact with infiltrating microglia via dynamic microglial processes. These contacts likely result in photoreceptor axon and process retraction, which is then followed by the phagocytosis of rod somata and the clearance of living cells (orange box). Application of the vitronectin receptor antagonist, cRGD, inhibits rod phagocytosis, and also decreases microglial activation, thus reducing the positive-feedback recruitment and activation of other nearby microglia. Reduction in microglial phagocytosis, via direct inhibition or via general microglial depletion, therefore results in decreased microglial clearance via this non-apoptotic mechanism. Activated, infiltrating microglia can additionally influence and potentiate the apoptotic route for rod death via IL-1β secretion, possibly via direct signaling and/or the indirect activation of Müller cells.

We have summarized these mechanisms underlying the influence of infiltrating microglia on non-cell-autonomous rod degeneration in the rd10 retina in a schematic (Fig9). Causative mutations initiate cellular stress in rods via cell-autonomous mechanisms, which generate the secretion of chemoattractive signals. These signals attract and recruit inner retinal microglia into the ONL, which come into close contact with photoreceptors. Recognizing “eat-me” signals on stressed rods, infiltrating microglia dynamically interact with and phagocytose living rods and become more activated in the process. These microglia upregulate activation markers and increase their secretion of pro-inflammatory IL-1β. IL-1β, either acting directly on rod photoreceptors (Scuderi *et al*, 2015), or indirectly via the pro-inflammatory activation of Müller cells (Liu *et al*, 2015), can potentiate rod stress and apoptosis, further driving microglial activation and infiltration in a positive-feedback manner. Maintained presence of phagocytic microglia in the ONL sustains the clearance of non-apoptotic rods, thereby accelerating the overall rate of rod demise. Evidence for this microglial phagocytosis of photoreceptors was found in multiple mouse models and human specimens of RP, underscoring it as a cell-death mechanism shared across different genetic etiologies of RP. While the presence of photoreceptor mutations constitutes the primary defect triggering cell-autonomous photoreceptor dysfunction in many retinal degenerative diseases, the maladaptive responses by retinal microglia to this initial event can additionally potentiate the rate of photoreceptor degeneration. Our findings here indicate that therapeutic strategies targeting retinal microglia may potentially be broadly applied to patients with RP across varied genetic etiologies and can be successful in prolonging the survival of endangered photoreceptors and in deferring irreversible vision loss.

## Materials and Methods

### Animals

Experiments were conducted according to protocols approved by a local Institutional Animal Care and Use Committee and adhered to the Association for Research in Vision and Ophthalmology (ARVO) Statement animal use in ophthalmic and vision research. The following strains of mice were obtained from The Jackson Laboratory (Bar Harbor, ME): mice homozygous for the *Pde6b*^*rd10*^ (rd10) mutation and the *Pde6b*^*rd1*^ (rd1), and CX3CR1^GFP/GFP^ and CCR2^RFP/RFP^ transgenic mice. Animals were housed in a National Institutes of Health animal facility under a 12-h light/dark cycle with food *ad libitum*. For *ex vivo* live imaging of microglial phagocytosis, rd10 mice were crossed with CX3CR1^GFP/GFP^ transgenic mice (Jung *et al*, 2000) to generate rd10, CX3CR1^+/GFP^ mice in which retinal microglia express green fluorescent protein (GFP) in a model of retinal degeneration. CX3CR1^GFP/GFP^ and CCR2^RFP/RFP^ transgenic mice were also crossed into the rd10 background to generate rd10, CX3CR1^+/GFP^, CCR2^+/RFP^ mice in which infiltrating systemic monocytes express red fluorescent protein (RFP) (Saederup *et al*, 2010). Transgenic mice in which the CX3CR1 gene was replaced by a sequence encoding a mutant Cre protein with a tamoxifen (TAM)-dependent estrogen ligand-binding domain (CX3CR1^CreER^) were employed as previous described (Parkhurst *et al*, 2013). These were crossed with mice containing a flox-STOP-flox diphtheria toxin subunit alpha (DTA) gene cassette in the ROSA26 locus (Jackson Laboratory, #009669) (Rosa26-flox-STOP-flox –DTA mice) (Voehringer *et al*, 2008). The progeny were crossed into the rd10 background to generate mice homozygous for the rd10 mutation and heterozygous for CX3CR1^CreER^ and DTA (rd10/CreDTA mice). Retinal microglia in these mice were depleted by the activation of Cre upon tamoxifen administration. The above mouse strains were maintained on a C57BL/6 background, and both male and female mice in the age range of postnatal days 18–50 were used as specified in individual experiments. Mice homozygous for the loss-of-function mutation in the CEP290 gene (rd16) and RPGRIP were obtained from Dr Tiansen Li (NEI).

### Human eye tissue

Adult human eyes with diagnosed RP were obtained from the donor programs of the Foundation Fighting Blindness (Columbia, MD). Retinal tissue was obtained from the following archived specimens: E-79-181, FFB-215, FFB-424, and FFB-316. Eyes were fixed with 2–4% paraformaldehyde, the retinas were dissected out and sectioned into 75-μm-thick vibratome sections. Eye tissue was collected under applicable regulations and guidelines with proper consent, protection of human subjects, and donor confidentiality.

### Immunohistochemistry and TUNEL labeling of retinal sections

Mice were euthanized by carbon dioxide inhalation and their eyes were enucleated and lenses removed. The resulting eyecups were marked as to their orientation and then fixed in 4% paraformaldehyde for 1 h at room temperature. Eyecups were embedded in 7% agarose and sectioned through the optic nerve in the superior-inferior plane into 100-μm-thick sections using a vibratome (VT1000, Leica). Sections were blocked and permeabilized (5% normal goat serum in 1× PBS with 0.5% Triton X-100 for 3 h at room temperature), and then incubated in primary antibodies in 1× PBS with 0.5% Triton X-100 for 36 h at 4°C. Primary antibodies included rabbit anti-Iba1 (Wako, #019-19741, 1:500), rabbit anti-PBR (TSPO) (Abcam, #ab109497, 1:200), rat anti-CD11b (AbD Serotec, #MCA711, 1:50), rat anti-CD68 (AbD Serotec, #MCA1957, 1:500), hamster anti-MFG-E8 (MBL, #D199-3, 1:250), rabbit anti-cleaved caspase 3 (Cell Signaling, #9661, 1:200), mouse anti-IL-1β (Cell Signaling, #12242S, 1:50), mouse anti-PARP (Enzo Life Technologies, #ALX-804-220, 1:100), mouse anti-PS (Millipore, #05-719, 1:250), rabbit anti-Cone Arrestin (Millipore, #AB15282, 1:100), rabbit anti-EEA1 (Abcam, #ab2900, 1:100), rabbit anti-rab5 (Cell Signaling, #2143S, 1:100), and mouse anti-Rhodopsin (Millipore, #MAB5356, 1:100). IL-1Triton X-100 was omitted in reactions involving antibodies to phosphatidylserine and MFG-E8, as previously performed (Mustafi *et al*, 2011; Neniskyte *et al*, 2011). After washing in 1× PBS with 0.5% Triton X-100, sections were incubated overnight with secondary antibodies (Alexa Fluor-488-conjugated goat anti-rabbit or rat IgG for Iba1 or CD11b, respectively; Alexa Fluor-568-conjugated goat anti-rabbit, mouse, hamster, or rat IgG for photoreceptor markers, PS, MFG-E8, or CD68, respectively) and DAPI (1:500; Sigma). Annexin V, conjugated to Alexa Fluor-568, was used as an additional marker for PS exposure (Life Technologies, #A13202, 1:250). Experiments in which primary antibodies were omitted served as negative controls. Apoptotic photoreceptors were labeled with a terminal deoxynucleotidyl transferase dUTP Nick End Labeling (TUNEL) assay (Roche, Indianapolis, IN) according to the manufacturer’s specifications. Stained retinal sections were imaged with confocal microscopy (FluoView 1000, Olympus). Multiplane z-series were collected using a 40× oil-immersion objective; each z-series spanned 20 μm in depth, with each section spaced 1 μm apart. Confocal image stacks were viewed and analyzed with FV100 Viewer Software (Olympus) and Image J (NIH).

### Live time-lapse confocal imaging of microglial phagocytosis

rd10, CX3CR1^+/GFP^ and rd10, CX3CR1^+/GFP^, CCR2^+/RFP^ mice used in these experiments were euthanized and immediately enucleated. The anterior segment and lens were dissected free, and the eyecups were placed in oxygenated Ringer’s solution (125 mM NaCl, 5 mM KCl, 1.5 mM CaCl_2_, 0.75 mM MgCl_2_/6H_2_O, 1.25 mM NaH_2_PO_4_, 10 mM D-glucose, 20 mM HEPES; pH 7.35–7.45). Retinal explants were dissected free from the eyecups and flat-mounted with the photoreceptor layer uppermost on Millipore filter paper (HABP045; Millipore, Billerica, MA, USA). Prior to live imaging, explants were incubated in an oxygenated chamber in Ringer’s solution containing propidium iodide (PI; 1:5,000; Life Technologies) to label apoptotic nuclei and Hoechst 33342 (1:500; Life Technologies) to label all ONL nuclei. Explants were then transferred to a 32°C temperature-controlled stage (Bioptechs, Butler, PA, USA) through which oxygenated Ringer’s solution was continuously superfused. Dynamic behavior of GFP-labeled microglia was followed with time-lapse imaging with a confocal microscope (FV1000, Olympus) using a 40× or 60× immersion objective. Z-series stacks of retinal microglia within the ONL were captured at a resolution of 1,024 × 1,024 pixels at 1-min time intervals over durations up to 2 h. A total of 30 time-lapse recordings involving eight retinas from four animals of the age range P21–24 were reviewed and analyzed.

### Microglial depletion in rd10 mice

In order to deplete microglia within the retina, rd10/CreDTA mice were orally gavaged with tamoxifen (Sigma) as a solution in corn oil (Sigma) (250 μl of a 20 mg/ml solution) as previously described (Parkhurst *et al*, 2013). Animals received an initial tamoxifen dose (5 mg) at P21–22, followed by a second equal dose 48 h later, followed by gavages every 5 days. Control animals were gavaged with corn oil without tamoxifen. Animals from the same litter were divided equally and randomized into the depleted group (treated with tamoxifen in corn oil) and the control group (treated with corn oil only) to decrease any systematic errors; analysis performed in a blinded fashion to assignment group. The experiments were performed independently nine times (*n* = 4 for P28–29, *n* = 2 for P37–39, and *n* = 3 for P50).

### Inhibition of phagocytosis with cRGD peptide administration

Microglial phagocytosis was inhibited using cyclic RGD (Arg-Gly-Asp-Phe-Val) peptide, an inhibitor of the vitronectin receptor, an αV integrin phagocytosis receptor, with its inactive analogue, cyclic RAD peptide (Arg-Ala-Asp-Phe-Val), as a negative control. In *ex vivo* experiments, acutely isolated retinal flat-mount explants from rd10/CX3CR1^+/GFP^ animals were incubated in Ringer’s solution containing cRGD or cRAD (400 μM, Bachem, Bubendorf, Switzerland), or in Ringer’s solution alone while being maintained in an oxygenated chamber. After 1 h, the explants were fixed, incubated with DAPI (1:500), and imaged with confocal microscopy. In *in vivo* experiments, cRGD and cRAD were injected intravitreally into rd10 mice at P20 (4 mM, 1.0–1.5 μl injection). Each experimental animal received cRGD in one eye and cRAD in the contralateral control eye; the order of eye (right or left) receiving cRGD was randomly assigned in experimental replicates. Animals were sacrificed at P23, 3 days following injection, and their retinas were analyzed. The experiments were performed independently twice.

### Inhibition of IL-1 signaling with recombinant IL-1RA

IL-1β signaling in the rd10 retina was inhibited using anakinra (Sobi, Stockholm, Sweden), a commercialized recombinant version of human IL-1 receptor antagonist. Each experimental rd10 animal received at P20–22 an intravitreal injection of anakinra in one eye (diluted in PBS, 1.0 μl injection; final vitreous concentration 60–150 μg/ml), and a control PBS injection of equal volume in the contralateral eye. A second anakinra/control injection was given 3 days later. Animals were sacrificed at P26–27 and their retinas were analyzed. The order of eye (right or left) receiving cRGD was randomly assigned in experimental replicates. For experiments involving intravitreal delivery of exogenous agents, animals that developed complications from the injection procedure (e.g., ocular infection, inflammation) were excluded from the analysis. This criteria was pre-established and involved < 5% of treated animals. The experiments were performed independently three times.

### Measurement of cytokine levels

Dissected mouse retinas were placed into 200 μl of protein lysate buffer (Complete Ultra, Roche) with proteinase inhibitor cocktail (Calbiochem, Gibbstown, NJ) at 4°C. Following homogenous and centrifugation, protein concentration was measured (BCA protein assay kit, Thermo, Pierce). Cytokine levels were determined using a Milliplex® assay kit (Milliplex MAP mouse cytokine/chemokine magnetic bead panel, #MCYTOMAG-70K, Millipore Corp., St. Charles, MO, USA) according to the manufacturer’s protocol using the Luminex 200TM (Luminex Corporation, Austin, TX). Data were analyzed using the Bio-Plex manager software (Bio-Rad Laboratories, Inc. Hercules, CA).

### Electroretinographic analysis

Electroretinographs (ERGs) were recorded in rd10 mice using an Espion E^2^ system (Diagnosys, Littleton, MA). Mice were dark-adapted overnight and prepared for recording in darkness under dim-red illumination. Mice were anesthetized with intraperitoneal ketamine (90 mg/kg) and xylazine (8 mg/kg), and were topically administered tropicamide (1%, Alcon) and phenylephrine (2.5%, Alcon) for pupillary dilation and proparacaine hydrochloride (0.5%, Alcon) for topical anesthesia. Flash ERGs recordings were obtained simultaneously from both eyes with gold wire loop electrodes, with the reference electrode was placed in the mouth and the ground subdermal electrode at the tail. ERG responses were obtained at increasing light intensities over the ranges of 1 × 10^−4^ to 10 cd·s/m^2^ under dark-adapted conditions, and 0.3 to 100 cd·s/m^2^ under a background light that saturates rod function. The stimulus interval between flashes varied from 5 s at the lowest stimulus strengths to 60 s at the highest ones. Two to 10 responses were averaged depending on flash intensity. ERG signals were sampled at 1 kHz and recorded with 0.3 Hz low-frequency and 300 Hz high-frequency cutoffs. Analysis of a-wave and b-wave amplitudes was performed using customized Espion ERG Data Analyzer software (v2.2) that digitally filters out high-frequency oscillatory potential wavelets. The a-wave amplitude was measured from the baseline to the negative peak, and the b-wave was measured from the a-wave trough to the maximum positive peak.

### Image analysis

Morphological analyses in sagittally oriented retinal sections traversing the optic nerve were performed consistently in the inferior mid-peripheral region of the retina at a distance 0.75 to 1.25 mm inferior to the optic nerve. Comparisons of retinal microglia, TUNEL^+^ nuclei, and rhodopsin- and cone-arrestin-labeled photoreceptors, were performed on z-projections of confocal stacks of uniform depth. Mean thickness measurements of the ONL was averaged over a 40× imaging field and calculated using image processing functions in ImageJ. Areas of CD68, MFG-E8, and PS immunopositivity were derived by thresholding images captured under uniform imaging conditions and expressed as a fraction of the area of the ONL (*n* ≥ animals per time point). In time-lapse imaging experiments, images were processed using ImageJ as previously described (Damani *et al*, 2011; Fontainhas *et al*, 2011). Briefly, two-dimensional time-lapse movies were created from maximum intensity projections in the *z*-dimension and aligned in the *x*–*y* plane using the StackReg plugin.

### Statistical analysis

The required sample sizes in animal experiments were calculated based on estimates of mean differences, variances, and power. All data were analyzed using statistical software (GraphPad Prism Software, Version 6.0.1). A normality test (D’Agostino and Pearson) was used to analyze the distribution of all data sets. For two-way comparisons of data following a Gaussian distribution, independent data sets were analyzed with an unpaired two-tailed *t*-test, whereas paired data sets (comparison of fellow eyes treated with cRGD vs. cRAD or anakinra vs. PBS control) were analyzed with a paired *t*-test. Correlation analysis was performed by the computation of the Spearman correlation. A *P*-value < 0.05 was set as the basis for rejecting the null hypothesis. In all graphical representations, the error bars indicate standard error (SE).

## Supporting Information

**Expanded View Figures PDF**

**Movie EV1**

**Movie EV2**

**Movie EV3**

**Movie EV4**

**Movie EV5**

**Movie EV6**

**Movie Legends**

**Review Process File**
