# Supplementary material for: Microglial phagocytosis of living photoreceptors contributes to inherited retinal degeneration
Source: EMBO Mol Med. 2015 Jul 2;7(9):1179–97. doi: 10.15252/emmm.201505298 (PMC4568951; doi:10.15252/emmm.201505298)
Supplement: Supplementary file 1 [file emmm0007-1179-sd1.pdf]

Expanded View Figures

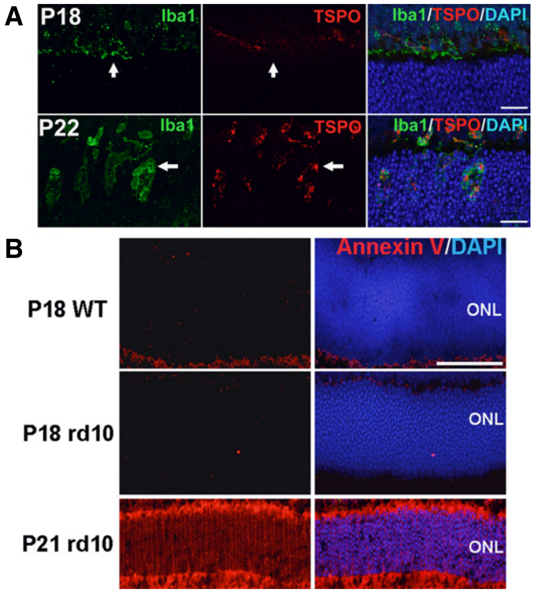

Figure EV1. Activation status of microglia infiltrating the outer nuclear layer (ONL) and the exposure of phosphatidylserine (PS) on ONL photoreceptors.

A Microglia infiltrating the outer nuclear layer (ONL) of the rd10 retina during rod photoreceptor degeneration demonstrate markers of activation. At P18, Iba1$^+$ microglia (green, arrow) in the outer plexiform layer were negative for TSPO (red), an activation marker. At P22, Iba1$^+$ microglia (arrow) infiltrated the ONL and acquired TSPO immunopositivity, indicating their activated status. Scale bars, 25 µm.

B Phosphatidylserine (PS) exposure in ONL nuclei of unfixed cryosections of rd10 retina during rod degeneration. PS exposure in the ONL was monitored in unfixed frozen sections using fluorescently conjugated annexin V which binds cell-surface PS. While minimal annexin V staining was evident in the ONL in P18 wild-type (top row) and P18 rd10 (middle row) retinas in which rod degeneration is absent, staining was prominent in P21 rd10 retina during rod degeneration (bottom row). Scale bar, 40 µm.

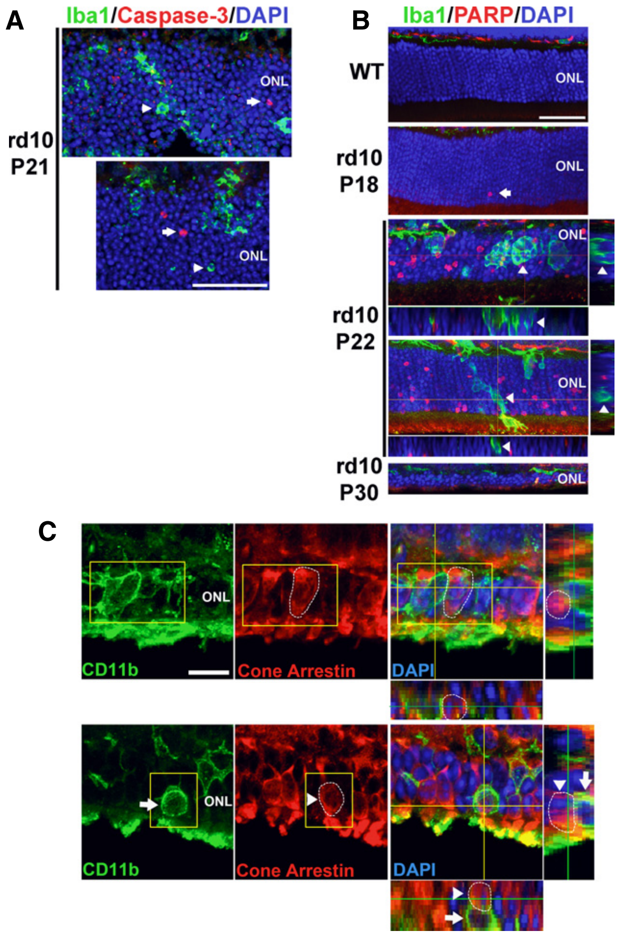

Figure EV2. Infiltrating microglia phagocytose rods that are immunonegative for markers of early apoptosis and do not phagocytose cones during rod degeneration.

A In P21 rd10 retina, ONL nuclei immunopositive for activated caspase-3 (red, arrows), an enzyme activated during early apoptosis, were found separately from phagocytized photoreceptor nuclei in microglial phagosomes (green, arrowheads). Scale bar, 40 µm.

B Immunohistochemistry for cleaved poly(ADP-ribose) polymerase (PARP), a substrate of activated caspase-3, shows the absence of immunopositive ONL nuclei in adult wild-type (WT) mouse. In the rd10 retina, immunopositive ONL nuclei were rare at P18, prevalent at P22, and decreased at P30, as expected from the progression of rod degeneration. At P22, ONL nuclei phagocytosed by microglia (arrows in insets) are predominantly immunonegative for cleaved PARP (arrowheads in orthogonal views). Scale bar, 40 µm.

C Photoreceptor cones are not phagocytosed by microglia during rod degeneration. (Upper panels) At P21–23, although infiltrating microglia in the ONL (CD11b, green) contain phagocytosed nuclei (DAPI, blue), none of these were found to be associated with cone arrestin immunopositivity (red), despite the close proximity of arrestin-positive cone somata (highlighted by circled area) to infiltrating microglia. (Lower panels) Example of a CD11b-positive microglial cell in the ONL juxtaposed closely to an arrestin-positive soma (highlighted by circled area). Analysis of orthogonal views of the confocal image stack demonstrates the absence of cone phagocytosis by microglia. Scale bar, 10 µm.

 © 2015 The Authors

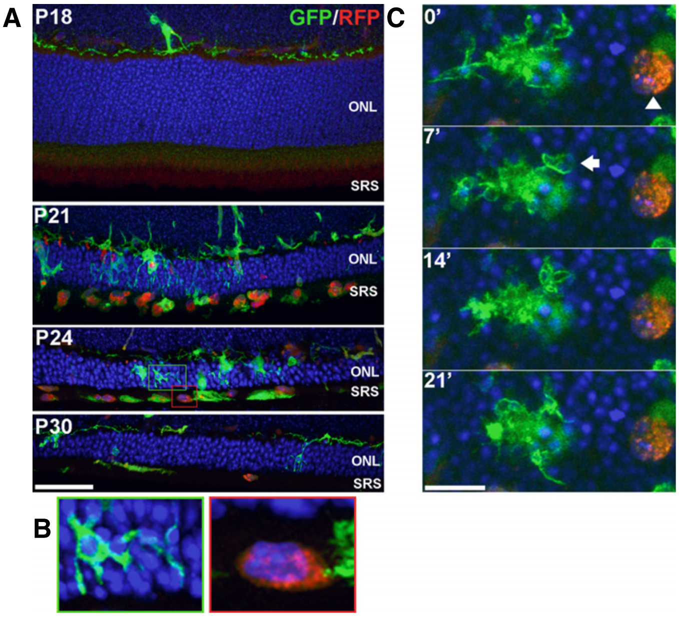

Figure EV3. Infiltrating retinal microglia, endogenous to the retina, primarily mediate photoreceptor phagocytosis, with little or no participation of systemically recruited monocytes.

A Retinas from transgenic CX3CR1$^{+/GFP}$, CCR2$^{+/RFP}$ mice crossed into the rd10 background were examined at time points before and during rod degeneration. At P18, prior to degeneration, the outer nuclear layer (ONL) and subretinal space (SRS) were relatively devoid of infiltrating cells. At P21, numerous GFP$^+$, RFP$^-$ retinal microglia infiltrate the ONL and engulf photoreceptors. Concurrently, a number of RFP$^+$ monocytes with rounded morphologies appeared predominantly in the SRS, with only a few cells within the ONL. At P24 and P30, RFP$^+$ monocytes remained concentrated in the SRS and declined in number with time. Scale bar, 40 μm.

B Morphological features of GFP$^+$, RFP$^-$ retinal microglia and RFP$^+$ monocytes differ significantly from each other. Infiltrating microglia (from green inset from P24 in A) demonstrated multiple processes contacting and engulfing photoreceptors in phagosomes, while RFP$^+$ monocytes (red inset) lacked processes, had limited contact with photoreceptors, and lacked evidence of intracellular phagosomes.

C Dynamic behavior also differed significantly between the two cell types as observed in a P24 retina explant by live confocal imaging: a GFP$^+$, RFP$^-$ retinal microglial cell (left) demonstrated typical dynamic process movement and engulfment (arrow), while RFP$^+$ monocytes (arrowhead) were relatively stationary and lacked dynamic process behavior. Scale bar, 10 μm.

© 2015 The Authors
