# Supplementary material for: Microglial phagocytosis of living photoreceptors contributes to inherited retinal degeneration
Source: EMBO Mol Med. 2015 Jul 2;7(9):1179–97. doi: 10.15252/emmm.201505298 (PMC4568951; doi:10.15252/emmm.201505298)
Supplement: Supplementary file 8 [file emmm0007-1179-sd8.docx]

**Expanded View Movie Legends**

**Movie EV1.** Movie corresponding to Figure 5A. Each frame is a z-merge of an image stack spanning the dimensions of the microglial cell. Frames are collected at the rate of 1 frame/min. Movie shows transient and repetitive contact between infiltrating ONL microglia and nearby photoreceptor somata using phagocytic “cups” at the termini of microglial processes.

**Movie EV2.** Movie corresponding to Figure 5B. Movie demonstrates the phagocytic interaction between microglia and photoreceptor soma (indicated by arrow) as comprised of (1) initial contact with phagocytic “cup”, (2) engulfment of soma, and (3) intracellular translocation photoreceptor towards microglial cell body. Transient contacts between microglial processes and photoreceptor somata (marked with *) occur concurrently with phagocytosis.

**Movie EV3.** Movie corresponding to Figure 5C. Movie demonstrates the engulfment of a photoreceptor soma via a lamellipodal microglial process and its subsequent translocation towards the microglial cell body where it is retained.

**Movie EV4.** Movie corresponding to Figure 5D. Movie demonstrates the engulfment of a photoreceptor soma (arrow) by an amoeboid microglia lacking elongated processes.

**Movie EV5 .** Movie demonstrates the migration of an amoeboid, phagosome-containing microglial cell through the ONL.

**Movie EV6.** Dynamic live cell imaging of infiltrating endogenous retinal microglia and recruited monocytes in the rd10 retina. Movie obtained from imaging of a retinal explant from a P24 CX3CR1^+/GFP^, CCR2^+/RFP^ rd10 mouse demonstrated dynamic process motility and photoreceptor contact and engulfment in an infiltrating retinal microglial cell located in the ONL (*left,* labeled with GFP and negative for RFP). In contrast, a nearby RFP+ monocyte (*right*) showed minimal motility and no phagocytic behavior.
