# Supplementary material for: Microglial phagocytosis of living photoreceptors contributes to inherited retinal degeneration
Source: EMBO Mol Med. 2015 Jul 2;7(9):1179–97. doi: 10.15252/emmm.201505298 (PMC4568951; doi:10.15252/emmm.201505298)
Supplement: Supplementary file 9 [file emmm0007-1179-sd9.pdf]

EMBO Molecular Medicine

Microglial phagocytosis of living photoreceptors contributes to inherited retinal degeneration

Lian Zhao, Matthew K. Zabel, Xu Wang, Wenxin Ma, Parth Shah, Robert N. Fariss, Hao-hua Qian, Christopher N. Parkhurst, Wen-Biao Gan, and Wai T. Wong

Corresponding author: Wai Wong, National Eye Institute

Review timeline:

Submission date:	30 March 2015
Editorial Decision:	16 April 2015
Revision received:	11 May 2015
Editorial Decision:	26 May 2015
Revision received:	28 May 2015
Accepted:	01 June 2015

Transaction Report:

(Note: With the exception of the correction of typographical or spelling errors that could be a source of ambiguity, letters and reports are not edited. The original formatting of letters and referee reports may not be reflected in this compilation.)

Editor: Céline Carret

1st Editorial Decision 16 April 2015

Thank you for the submission of your manuscript to EMBO Molecular Medicine. We have now heard back from the three referees whom we asked to evaluate your manuscript.

You will see that all three referees find your article to be of great interest and while referees 1 and 2 only have a few suggestions to increase the impact of the paper and its conclusiveness, referee 3 is more critical. We would like to ask you to reply in details to referee 3 concerns. If you already have data addressing the main issue raised by this referee about whether defective microglia may be the initial driving event in the retinal degeneration process, please add them to the revised manuscript, but we will not consider this as a precondition for the re-review of a revision.

Given these evaluations, we would welcome the submission of a revised version for further consideration and depending on the nature of the revisions, this may be sent back to the referees for another round of review.

Please note that it is EMBO Molecular Medicine policy to allow only a single round of revision and that, as acceptance or rejection of the manuscript will depend on another round of review, your responses should be as complete as possible.

EMBO Molecular Medicine has a "scooping protection" policy, whereby similar findings that are published by others during review or revision are not a criterion for rejection. Should you decide to submit a revised version, I do ask that you get in touch after three months if you have not completed it, to update us on the status.

Please also contact us as soon as possible if similar work is published elsewhere. If other work is published we may not be able to extend the revision period beyond three months.

I look forward to receiving your revised manuscript.

***** Reviewer's comments *****

Referee #1 (Comments on Novelty/Model System):

The paper is clearly written and the experiments were well thought out. These experiments include evaluating mouse models of RD and corroborating with human RP samples.

I have suggested to the authors that the use of IHC to demonstrate phagosomes such as Eea1 would strengthen their study.

Otherwise, this study will be of great interest to investigators studying retinal diseases and/or immunology.

Referee #1 (Remarks):

The authors present evidence that microglia engulf photoreceptor nuclei concomitant with increased expression of 'eat-me' signals on rods carrying the rd10 mutation. They present beautiful videos of microglial interactions with photoreceptor nuclei, and histological and visual function measurements in mouse models in which microglial phagocytosis has been inhibited, to support the role of microglia in phagocytosis. The study is nicely done and the paper is well written.

I have the following recommendation:
The demonstration of 'multiple phagasomes' as indicated on Page 5, end of first paragraph of results section (and throughout the manuscript), would ideally involved phagasome markers, otherwise this is descriptive and it is not clear. Markers such as Eea1 and rab5 may be used for IHC.

Referee #2 (Remarks):

The authors assess the role of microglia in loss of rod cells in a model of Retinitis pigmentosa, and conclude that microglia contribute via phagocytosis of stressed-but-viable rod cells and via IL-1b-induced apoptosis. The work is well motivated, designed and carried out, and comes to an important and novel conclusion in a convincing way. Multiple, complimentary approaches are used. The date are well, described, analysed and presented. Overall I think this an excellent paper. I have one a few minor comments/questions.
1. Lactoferrin should Lactadherin.
2. I am not convinced that is possible to measure phosphatidylserine exposure of cells in fixed or cryopreserved sections. Phosphatidylserine go from the inside to outside of plasma membrane during stress or apoptosis. But an antibody to PS or Annexing V should have access to both sides of the membrane in cryopresereved tissue as the plasma membrane is unlikely to be intact.
3. Is it really likely that IL-1beta would induce apoptosis directly in rod cells rather than increasing the activation of microglia, astrocytes or other cells?

Referee #3 (Comments on Novelty/Model System):

Microglial activation in retinal disease is a sine qua non and is information now in the text books. The authors have not made the case that this is the primary defect in their retinal degeneration model.

Referee #3 (Remarks):

As before, this revised paper is a beautifully performed study on the removal of degenerating photoreceptors by microglia in mice with the RD10 mutation. The authors contend that the pathophysiological defect lies in the microglia which may drive the degeneration autonomously. However, they do not provide the evidence. As they state in last paragraph of the Results section, the 'mutation bearing rods in the rd10 retina specifically present a phagocytic "eat me" signal during the onset of rod degeneration'. The imagein Figure 2G confirms the extensive evidence of this signal at p22 when microglial presence and activation in this layer is at an early stage. The logical interpretation of these data is that the microglia are responding to this signal and arrive in this layer form the inner retina with the object of clearing the degenerating photoreceptor cells. This is the recognised physiological function of microglia. In the absence of microglia, less photoreceptor cells are removed as the authors also show in their conditional knock out experiments, but it is unlikely that these degenerating photoreceptors are fully functional.

1st Revision - authors' response 11 May 2015

Response to Reviewer #1

The authors present evidence that microglia engulf photoreceptor nuclei concomitant with increased expression of 'eat-me' signals on rods carrying the rd10 mutation. They present beautiful videos of microglial interactions with photoreceptor nuclei, and histological and visual function measurements in mouse models in which microglial phagocytosis has been inhibited, to support the role of microglia in phagocytosis. The study is nicely done and the paper is well written.

We appreciate the reviewer's comments and encouragements on the manuscript.

The demonstration of 'multiple phagasomes' as indicated on Page 5, end of first paragraph of results section (and throughout the manuscript), would ideally involved phagasome markers, otherwise this is descriptive and it is not clear. Markers such as Eea1 and rab5 may be used for IHC.

In our manuscript, we have employed CD68, which is also known as lysosomal/endosomal-associated membrane glycoprotein-(LAMP)-4, as the phagosome marker for the purpose indicated by the reviewer. Mouse CD68, also called macrosialin, is an 110kD glycoprotein that (1) shares significant homology with other lysosomal associated glycoproteins (PMID: 8486654), (2) localizes predominantly to late endosomal and lysosomal compartments (PMID: 10648004), and (3) has been implicated as a receptor for scavenging PS-rich liposomes (PMID: 7568176) and (4) as a lysosomal marker in activated microglia during neurodegeneration (PMID: 24948809).

In our results, we show that infiltrating microglia in the ONL at P22 develop CD68 immunopositivity (Fig. 2A) that co-localizes to vesicular structures within ONL microglia, identifying these structures as phagosomes (Fig. 2B). We also found that these CD68-positive phagosomes contain photoreceptor nuclei that are immunopositive also for rhodopsin (Fig. 3C), indicating that these phagosomes are involved with rod phagocytosis.

We have clarified this in the results (p. 5-6):

"As the morphological changes in infiltrating microglia suggested phagocytic function, we examined microglia for the expression of molecular markers of phagocytosis by immunohistochemistry. CD68, a lysosome-associated membrane protein (LAMP) and scavenger receptor (Holness et al, 1993), and a marker for activated phagocytic monocytic cells (Graeber et al, 1990), was expressed at very low levels prior to microglial infiltration at P18 (Fig. 2A), but increased prominently in the ONL at P21-22, co-localizing to Iba1+ infiltrating microglia, specifically to microglial phagosomes (Fig. 2B), before diminishing at P30 (Fig. 2C). This indicated that the vesicular structures located in ONL microglia were functional phagosomes."

We have also obtained some corroborative data that we have not included in the current manuscript. We found that microglial vesicular structures were also variably immunopositive for lysosomal-associated membrane protein (LAMP)-2 (also known as CD107b), a phagolysosomal protein (PMID: 17506821, 20404078) that has previously been found in activated macroglia/microglia in human brains with multiple system atrophy (MSA) (PMID: 22343695) and in an endotoxin mouse model of brain inflammation (PMID: 23758980). This data agrees with our data obtained with CD68 immunohistochemistry and confirms the vesicular structures in ONL microglia as phagosomes.

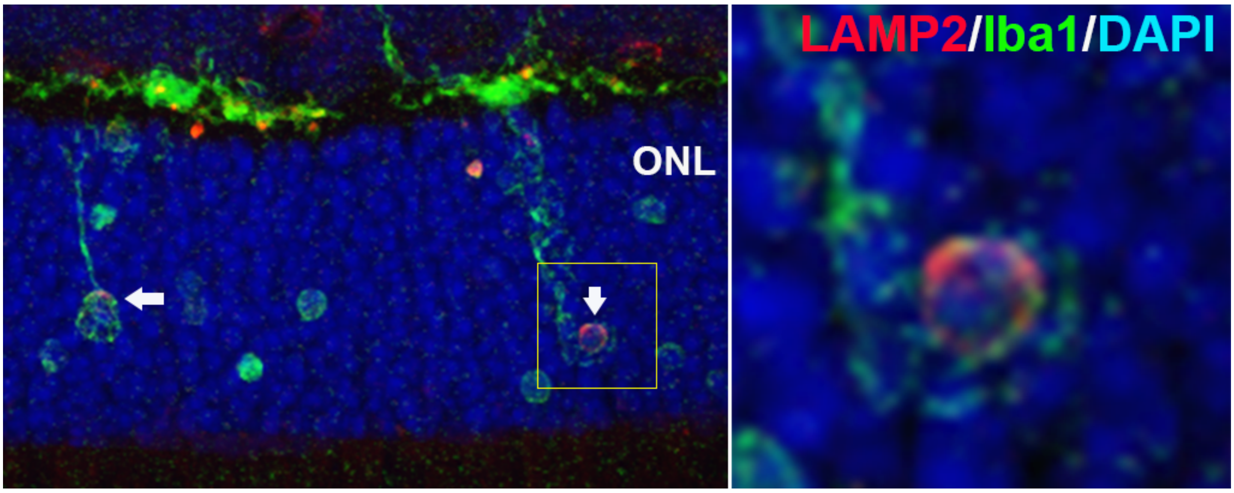

Response to Reviewer #2

The authors assess the role of microglia in loss of rod cells in a model of Retinitis pigmentosa, and conclude that microglia contribute via phagocytosis of stressed-but-viable rod cells and via IL-1b-induced apoptosis. The work is well motivated, designed and carried out, and comes to an important and novel conclusion in a convincing way. Multiple, complimentary approaches are used. The data are well, described, analysed and presented. Overall I think this an excellent paper.

We are grateful for the reviewer's positive assessment of our paper.

I have one a few minor comments/questions.

Lactoferrin should Lactadherin

We apologize for the error and have corrected it (p6).

I am not convinced that is possible to measure phosphatidylserine exposure of cells in fixed or cryopreserved sections. Phosphatidylserine go from the inside to outside of plasma membrane during stress or apoptosis. But an antibody to PS or Annexin V should have access to both sides of the membrane in cryopreserved tissue as the plasma membrane is unlikely to be intact.

We had performed PS immunohistochemistry following brief fixation in paraformaldehyde in the absence of detergent to avoid the loss of lipids and membrane breakdown. Variants of this protocol have been previously employed in other studies to detect the presentation of cell-surface PS (PMID: 21659555, with low detergent concentration (0.3% Triton); PMID: 21903584, with omission of detergent). We have referenced these in the Methods section (p20). In data we presented in Figure 2G, we did not find PS-immunopositivity in similarly-treated P18 retina prior to the onset of degeneration (which served as a negative control), giving us the assurance that the PS-immunopositivity we detected at P22 reflected actual PS exposure. To corroborate this result, we performed cell-surface detection of PS using fluorescently conjugated Annexin V (which binds exposed PS) in unfixed frozen retinal sections (cryosections) (Figure EV1B). In our negative

controls, which included (a) P18 wild type retina and (b) P18 rd10 retina (prior to rod degeneration), we detected minimal Annexin V in ONL nuclei, while in the ONL of P21 rd10, Annexin staining was prominent. These data taken together give credence to our observations regarding PS localization.

Is it really likely that IL-1beta would induce apoptosis directly in rod cells rather than increasing the activation of microglia, astrocytes or other cells?

The reviewer raises a relevant question on the cellular mechanism/s by which IL-1beta may potentiate apoptosis in rods: either (1) directly via signaling to rods or (2) indirectly via activation of microglia or macroglia. While our results do not demonstrate this, there is evidence in the literature that direct signaling to rods is possible. The receptor mediating IL-1β pro-inflammatory signaling, IL-1RI, is expressed broadly on retinal neurons, including photoreceptors in the ONL, and this receptor is found to be upregulated in pathological situations (e.g. diabetes) (PMID: 25676032). A recent study found that when IL-1β-expressing monocytes are cultured transiently (18h) in juxtaposition with photoreceptors in retinal flat-mounts, photoreceptor apoptosis can be induced in an IL-1β-dependent manner (PMID: 25948251), also suggesting a direct signaling mechanism.

Indirect signaling as suggested by the reviewer can also contribute. Exogenous IL-1β when applied to cultured retinal Müller cells can induce increased IL-6 expression (PMID: 25239226) and decreased glutamine synthetase (PMID: 19839866), indicating Müller cell activation in direct response to IL-1β.

The ability of microglia to themselves respond to IL-1β signaling in an autocrine fashion is however less well established. A recent study using a transgenic mouse reporter of IL-1RI expression did not detect expression in brain microglial cells (or astrocytes) under uninjured conditions (PMID: 25698726). Genetic deletion of IL-1R1 also did not inhibit the early activation of microglia in a model of trimethyltin-induced model of brain injury (PMID: 18191113). Though incompletely conclusive, these studies hint that autocrine IL-1β signaling onto microglia may not be a major factor in retinal degeneration.

To refer to the possibility of both direct and indirect IL-1β signaling mechanisms, we have amended the Discussion to include (p17):

"These microglia upregulate activation markers and increase their secretion of pro-inflammatory IL1β. IL1β, either acting directly on rod photoreceptors (Scuderi et al, 2015), or indirectly via the pro-inflammatory activation of Müller cells (Liu et al, 2015), can potentiate rod stress and apoptosis, further driving microglial activation and infiltration in a positive feedback manner."

And also in the legend of Figure 9 (p42):

"Activated, infiltrating microglia can additionally influence and potentiate the apoptotic route for rod death via IL-1β secretion, possibly via direct signaling and/or the indirect activation of Müller cells."

Response to Reviewer #3

Microglial activation in retinal disease is a sine qua non and is information now in the text books. The authors have not made the case that this is the primary defect in their retinal degeneration model.

The reviewer is correct in pointing out that microglial activation in the context of retinal disease is a well-established observation. We have provided a number of references in the Introduction that had previously noted this in the context of photoreceptor degeneration. However, whether microglial activation worsens or ameliorates photoreceptor degeneration is less clear. The cellular mechanisms by which microglia exert these non-cell autonomous effects are also yet undefined and as such constitute the central focus of the manuscript.

We do not intend to make the case that the primary defect in the rd10 model lays in retinal microglia. The initial trigger for rod apoptosis in the rd10 mouse is the presence of the rod-specific

PDE6b mutation and its cell-autonomous consequences; when this mutation is absent (as in wild type mice), microglial activation does not occur. We do however make the case that once rod injury secondary to the cell-autonomous effects of the mutation commences microglial cells are alerted and subsequently migrate into the ONL to interact dynamically with rod photoreceptors. These microglia-photoreceptor interactions contribute additionally to rod demise through microglia-mediated phagocytosis and pro-inflammatory influences. When microglia are depleted from the rd10 retina, rod degeneration is slowed (though not completely prevented), demonstrating the contribution that microglia make to potentiating the dynamics of rod demise.

As before, this revised paper is a beautifully performed study on the removal of degenerating photoreceptors by microglia in mice with the RD10 mutation.

We appreciate the reviewer's comment on the quality of our study.

The authors contend that the pathophysiological defect lies in the microglia which may drive the degeneration autonomously. However, they do not provide the evidence. As they state in last paragraph of the Results section, the 'mutation bearing rods in the rd10 retina specifically present a phagocytic "eat me" signal during the onset of rod degeneration'. The image seen in Figure 2G confirms the extensive evidence of this signal at p22 when microglial presence and activation in this layer is at an early stage. The logical interpretation of these data is that the microglia are responding to this signal and arrive in this layer form the inner retina with the object of clearing the degenerating photoreceptor cells. This is the recognized physiological function of microglia. In the absence of microglia, less photoreceptor cells are removed as the authors also show in their conditional knock out experiments, but it is unlikely that these degenerating photoreceptors are fully functional.

We agree with the reviewer's assessment that (1) microglial activation and infiltration into the ONL and (2) the presentation of PS, a "eat-me" signal on rods, occur concurrently at around P22. These infiltrating microglia do indeed play a role of clearing apoptotic rods, as evidenced by examples of microglia phagocytizing TUNEL+ photoreceptors (Fig 3H). This has been the expected function of microglia as pointed out by the reviewer. The novel finding in this manuscript however is the additional evidence that microglia also phagocytize stressed, mutation-bearing rods, which have not yet progressed to apoptosis. These mutation-bearing but living rods demonstrate residual rod function, albeit at levels below that of wild type mice. This is evidenced in the general clinical phenotype of human RP; while affected patients are born bearing the rod mutation; they are not born blind but only gradually lose rod function in the first few decades of life as the degeneration progresses with time. This is also true in rd10 mice – while they are born with the Pde6b mutation in their rods, ERG amplitudes (recorded from P18 to P26) progressively decrease with increasing age in parallel with the progression of rod degeneration (PMID: 17111372).

As such, the ability to slow down the overt degeneration of mutation-bearing rods by targeting the factors that drive it forward is of practical concern, as this can potentially defer vision loss in affected patients. Our data demonstrates that the depletion of microglia (Fig. 6) while not completely arresting rod loss, can delay rod demise by decreasing microglial phagocytosis and pro-inflammatory influences. We do not interpret the amelioration of ONL atrophy simply as the "piling up" of uncleared apoptotic cells for two reasons: (1) the density of TUNEL+ nuclei in the ONL was found to decrease, not increase, with microglial depletion (Fig. 6J, N, R), and (2) the amplitude of ERG responses, quite still below wild-type levels, was found to be better preserved with microglia depletion - demonstrating slowed degeneration as indicated by the documented natural history of ERG amplitude decline in rd10 mice (PMID: 17111372).

To better stress the contributory, as opposed to primary, role of microglia in the manuscript, we have made these changes in the following key areas of the manuscript:

Abstract (p2):

"Our findings highlight primary microglial phagocytosis as a ~~central~~ contributing mechanism underlying cell death in retinitis pigmentosa and implicate microglia as a potential cellular target for therapy."

Final paragraph of discussion (p17):

"While the presence of photoreceptor mutations constitutes the primary defect triggering cell-autonomous photoreceptor dysfunction in many retinal degenerative diseases, the maladaptive responses by retinal microglia to this initial event can additionally potentiate the rate of

photoreceptor degeneration. Our findings here indicate that therapeutic strategies targeting retinal microglia may potentially be broadly applied to RP patients across varied genetic etiologies and can be successful in prolonging the survival of endangered photoreceptors and in deferring irreversible vision loss."

2nd Editorial Decision 26 May 2015

Thank you for the submission of your revised manuscript to EMBO Molecular Medicine. We have now received the enclosed report from the referee asked to re-assess it. As you will see this reviewer is now supportive and I am pleased to inform you that we will be able to accept your manuscript pending some final editorial amendments.

Please submit your revised manuscript within two weeks.

I look forward to reading a new revised version of your manuscript as soon as possible.

***** Reviewer's comments *****

Referee #3 (Comments on Novelty/Model System):

This is my third review of this paper. Please see my previous concerns in review files.
The authors and I are now in agreement regarding the interpretation of their data, i.e. that the primary defect lies in the photoreceptors and that microglial activation is secondary but may contribute to the continuing decline in photoreceptor function.

Referee #3 (Remarks):

The authors have now revised their manuscript to provide good interpretation of their data.

© EMBO
